# Supplementary material for: Allele-specific DNA methylation is increased in cancers and its dense mapping in normal plus neoplastic cells increases the yield of disease-associated regulatory SNPs
Source: Genome Biol. 2020 Jun 29;21:153. doi: 10.1186/s13059-020-02059-3 (PMC7322865; doi:10.1186/s13059-020-02059-3)
Supplement: Supplementary file 2 — Additional file 2: Figure S1. Flow charts and diagram of computational and analytical approaches in this study. Figure S2. Summary of sample types and numbers and yield of informative SNPs. Figure S3. PCA of the combined WGBS and SureSelect methyl-seq data and series-wide overlap between ASM loci detected by the two methods. Figure S4. Distribution of ASM shows a high proportion of rare or private ASM in both cancer and normal samples and a significant increase in per-sample ASM in the cancers. Figure S5. Comparison of results from individual DNA samples analyzed by two WGBS library construction kits at two sequencing facilities, or by SureSelect and WGBS. Figure S6. Example of a chromosome region illustrating consistency between SureSelect methyl-seq, WGBS, and targeted bisulfite sequencing. Figure S7. Validations of ASM DMRs in disease-associated chromosomal regions: rs10411630 and multiple sclerosis. Figure S8. Validation of ASM DMRs in disease-associated chromosomal regions: rs2427290 and colorectal cancer. Figure S9. Validation of ASM DMRs in disease-associated chromosomal regions: rs2283639 and non-small cell lung carcinoma. Figure S10. Validations of ASM DMRs spanning a range of ASM ranks. Figure S11. Kernel density plots of methylation levels showing global hypomethylation and decrease in the percentage of highly methylated CpGs in cancers. Figure S12. Replication of the findings using WGBS from a single facility. Figure S13. Allele-specific losses of methylation leading to ASM in cancers. Figure S14. Kernel density plots of methylation level distributions showing statistically enriched instances of allele-specific gains of methylation in cancers. Figure S15. Shared ASM loci in cancer and non-cancer have similar ASM magnitude. Figure S16. Correlations between allelic TF binding affinity scores and ASM magnitude in the 4 classes of ASM loci. Figure S17. Examples of ASM DMRs in chromatin deserts. Figure S18. Models for inter-individual variability and allele-s [file 13059_2020_2059_MOESM2_ESM.pdf]

Fig. S1A, B. Flow charts and diagram of computational and analytical approaches in this study.

A, (Left panel) Steps for ASM calling and ranking, including ASM definition and criteria (See Methods). Our ASM definition incorporates both individual CpG and DMR-wide (multiple CpG) statistical criteria. (Right panel) Analytical pipeline for post-calling annotation and analyses to test ASM mechanisms, comparing ASM sub-classes (cancer vs non-cancer; desert vs non-desert), and overlaying that information with public GWAS data to nominate disease associated rSNPs and disrupted TF binding motifs. **B,** diagram of ASM criteria utilized in this study.

Fig. S1A

Identifying and ranking ASM DMRs

Sequence alignment to the reference methylome

- BisMark - default settings with PE mode (WGBS, Agilent) and SE mode (Agilent unpaired reads after trimming).

Heterozygous SNP calling on bisulfite-seq

- BisSNP - with Quality Score recalibration and maximum coverage less then 200x.
- Non-G/A SNP coverage > 5x per allele (total coverage > 10x)
- Coverage of allele B between 20% and 80% of total coverage
- Filter out false calls : SNPs with multiple alignment, > 2 alleles with AF>0.01, indels, no AF (UCSC browser annotation of dbSNP147)
- Filter out false calls: SNPs with in HW disequilibrium (exact FDR<0.05) and het. freq > expected het. freq (dbSNP147)

Identification of CpGs with ASM

- CpG coverage > 5X per allele
- Filter out CpGs destroyed by common SNPs (> 5% MAF)
- Filter out CpGs within 10 bp of PE read 2 for Nextera ("fill-in" region) and 7 bp of both reads for TruSeq
- Fisher exact test comparing methylation on allele A vs B ($p < 0.05$)
- Check predicted differences in methylation in AA vs. BB homozygotes using an mQTL-like approach

Identification & ranking of ASM DMRs

- Estimate DMR border (first and last ASM CpG) and count the # of significant CpGs in the DMR
- Compare methylation between alleles across the DMR: avg methylation across all covered CpGs between the first and last ASM CpG of the same DMR.
- **<u>Final criteria for calling ASM</u> : DMR difference >20% and BH-corrected Wilcoxon p-value < 0.05, <u>and</u> at least 3 ASM CpGs including at least 2 consecutive ASM CpGs (overlapping DMRs merged) – <u>see Fig. S1B</u>**
- Exclude DMRs in known imprinted chromosomal regions
- Rank DMRs by absolute methylation difference, number and percentage of ASM CpGs
- Independent validations by targeted bis-seq on a set of ASM loci with strong and weak ranks

Testing mechanisms in normal and cancer ASM; nominating disease-associated rSNPs

Functional annotation and enrichment analyses of features in ASM DMRs

- eQTLs; DNAse-HS, TF binding (ChIP-seq)
- Disrupted TFBS motif occurrences overlapping a cognate ChIP-seq peak (200 bp window)
- TF peaks for which the motif is enriched (≥10 fold) compared to background based on ENCODE ChIP-seq

Identification of TFBS motifs with disruptive SNPs that correlate with ASM

- Identify polymorphic TF motifs occurrences using ENCODE and JASPAR PWM and AtSNP software (require DNAse-HS peaks)
- Test for enrichment of disrupted vs non-disruptive polymorphic TF occurrences among ASM DMRs
- Test for correlations of ASM strength with PWM scores

Assess mechanistic similarities and differences between cancer and non-cancer ASM

- Multivariate analysis to compute odds ratios of finding ASM DMRs from cancer and non-cancer samples in specific chromatin states and associated with SNPs that disrupt specific TF binding motifs
- Assess rates of allele switching in cancer vs non-cancer ASM loci
- Assess frequencies of chromatin desert locations for cancer vs non-cancer ASM loci

Identification of hap-ASM DMRs in haplotype blocks that contain GWAS peaks

- Determine stringent and lenient LD and haplotype blocks
- Require distance between ASM and GWAS SNP < 200 kb
- Annotate ASM index SNPs for associations with immune/inflammatory, neuropsychiatric and neurodegenerative, neoplastic, and cardiometabolic diseases and traits

Creation of genome browser tracks for visualization and prioritization of candidate disease-associated rSNPs

- Custom tracks of ASM for each chromosome in UCSC Genome Browser format; tracks provide multiple annotations of each ASM index SNP.
- Annotations include ranking based on ASM strength and mechanistically relevant features including the identities of enriched and correlated TF and CTCF binding motifs that are disrupted by each ASM index SNP

Fig. S1B

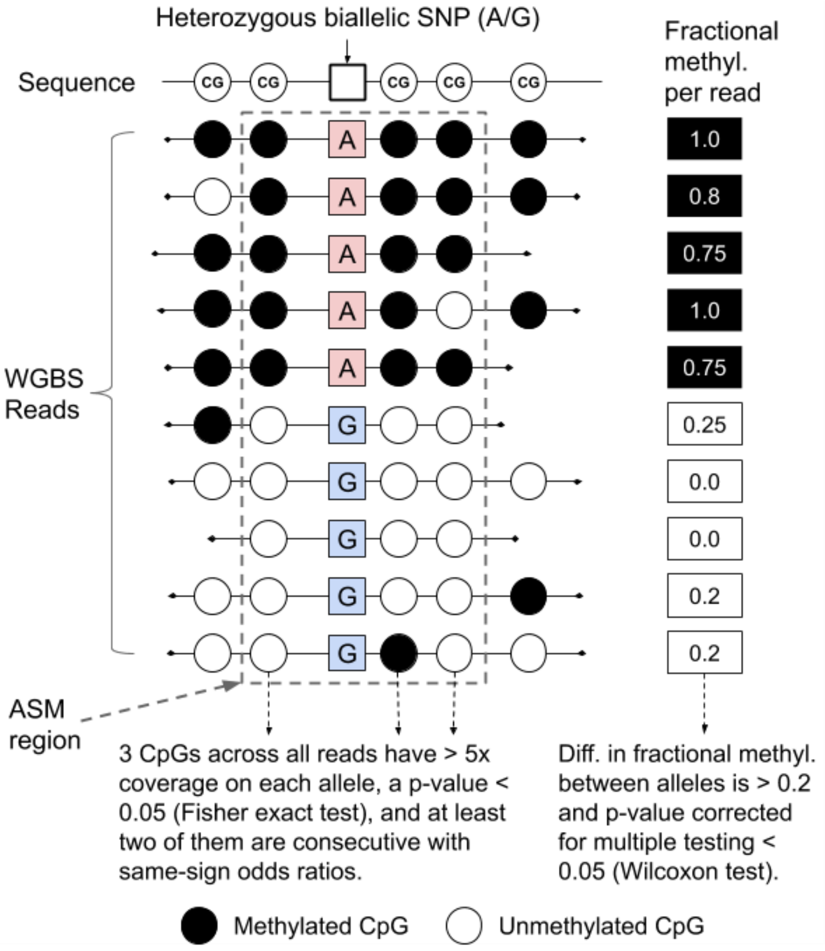

Heterozygous biallelic SNP (A/G)
Fractional methyl. per read
Sequence
CG
WGBS Reads
A
G
1.0
0.8
0.75
1.0
0.75
0.25
0.0
0.0
0.2
0.2
ASM region
3 CpGs across all reads have > 5x coverage on each allele, a p-value < 0.05 (Fisher exact test), and at least two of them are consecutive with same-sign odds ratios.
Diff. in fractional methyl. between alleles is > 0.2 and p-value corrected for multiple testing < 0.05 (Wilcoxon test).
Methylated CpG
Unmethylated CpG

Fig. S2. Summary of sample types and numbers and yield of informative SNPs

A, Summary of samples sequenced by Agilent SureSelect and WGBS. Additional information is in **Additional file 1: Table S1**. Since our high confidence ASM set required ASM in at least 2 samples, the final informative SNP set used for downstream analyses corresponds to the 2,485,759 SNPs that were informative in at least two samples. The Venn diagrams are schematic, not drawn to scale. The percentages are calculated on the union of Agilent and WGBS SNPs. In other words, 1% of the total number of informative SNPs in our dataset were found only in SureSelect samples, 3% are found in both WGBS and SureSelect and 96% only in WGBS samples, which means that 4% (3 + 1) of the SNPs were informative in SureSelect and 99% (96 + 3) in WGBS. **B**, Map of a region of chromosome 20, showing an increased yield of ASM SNPs in WGBS compared to SureSelect, as expected based on genomic coverage and a greater number of samples and therefore informative

Fig. S2

A

Agilent SureSelect (25 samples)

- 1 lymphoblastoid cell line
- 24 primary non cancer tissues/cells

9 brain cortex, 1 fetal lung, 2 fetal hearts, 1 fetal placenta, 2 livers, 3 PBL, 6 T cells

WGBS (81 samples)

- 5 normal cell types from explants grown in tissue culture (*2 bladder epith. cell lines, 3 mammary epith. cell lines)*
- 1 lymphoblastoid cell line
- 59 primary non-neoplastic tissues or purified cell types (3 *brain cortex, 4 glia, 5 NeuN+ neurons, 9 B cells, 13 CD3+ T cells, 2 CD4+, 2 CD8+, 2 macrophage preps, 7 monocytes, 2 PBL, 1 LN, 1 fetal side placenta, 1 fetal side CTB, 1 maternal side CTB, 1 maternal side EVT, 3 livers, 2 breast tissue*)
- 16 primary cancers (7 *multiple myeloma, 2 DBCL, 1 FL, 6 GBM)*

Informative SNPs

Agilent WGBS

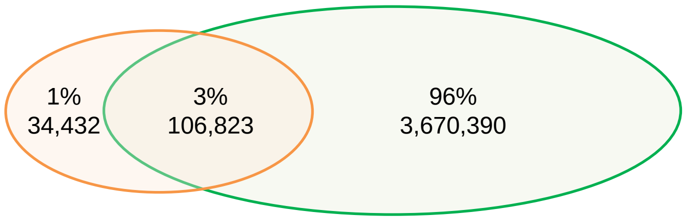

Informative SNPS in ≥ 2 biological samples

Agilent WGBS

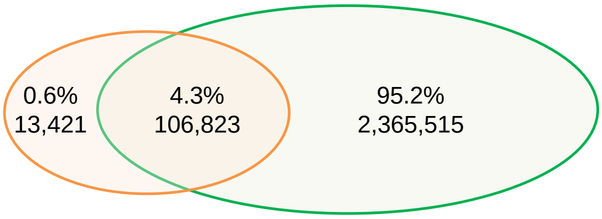

Percentage are calculated on the union of Agilent and WGBS SNPs

B

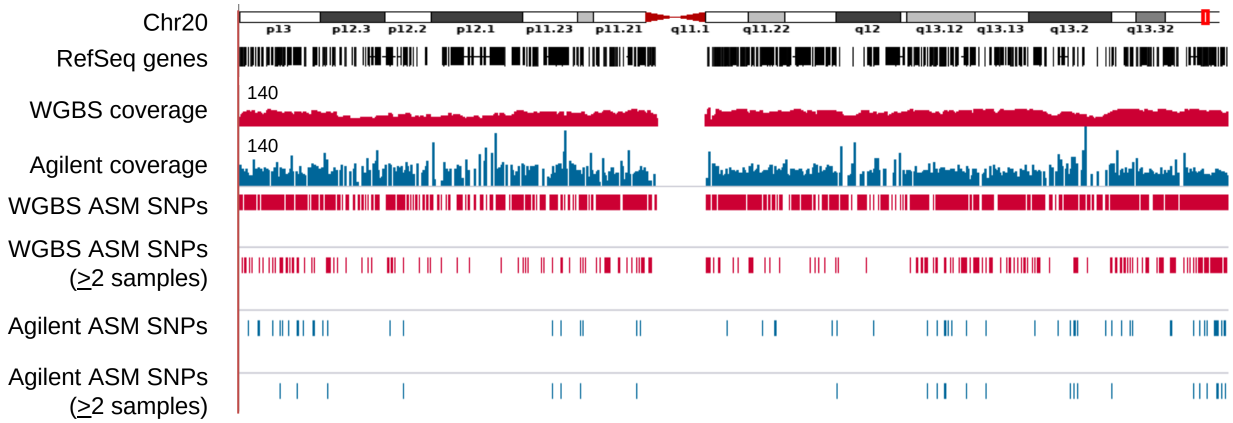

Fig. S3. PCA of the combined WGBS and SureSelect methyl-seq data and series-wide overlap between ASM loci detected by the two methods

A, PCA performed using net methylation values of CpGs on chromosome 20. Only CpGs informative (>10X) in both Agilent SureSelect and WGBS were used. The PCA shows clear clustering by cell/tissue and cancer type. Similar results were found using methylation data from other autosomes. **B**, Pie chart showing the proportion of high confidence ASM SNPs found in more than two biological samples, identified by WGBS and by SureSelect methyl-seq. Numbers of ASM SNPs are in parenthesis. **C**, Venn diagram showing a cross-platform comparison, with the percentage of high confidence ASM index SNPs that were identified series-wide in both assays. Only SNPs informative in both assays (adequate sequence coverage and heterozygous genotype calls) were considered for this comparison.

Fig. S3

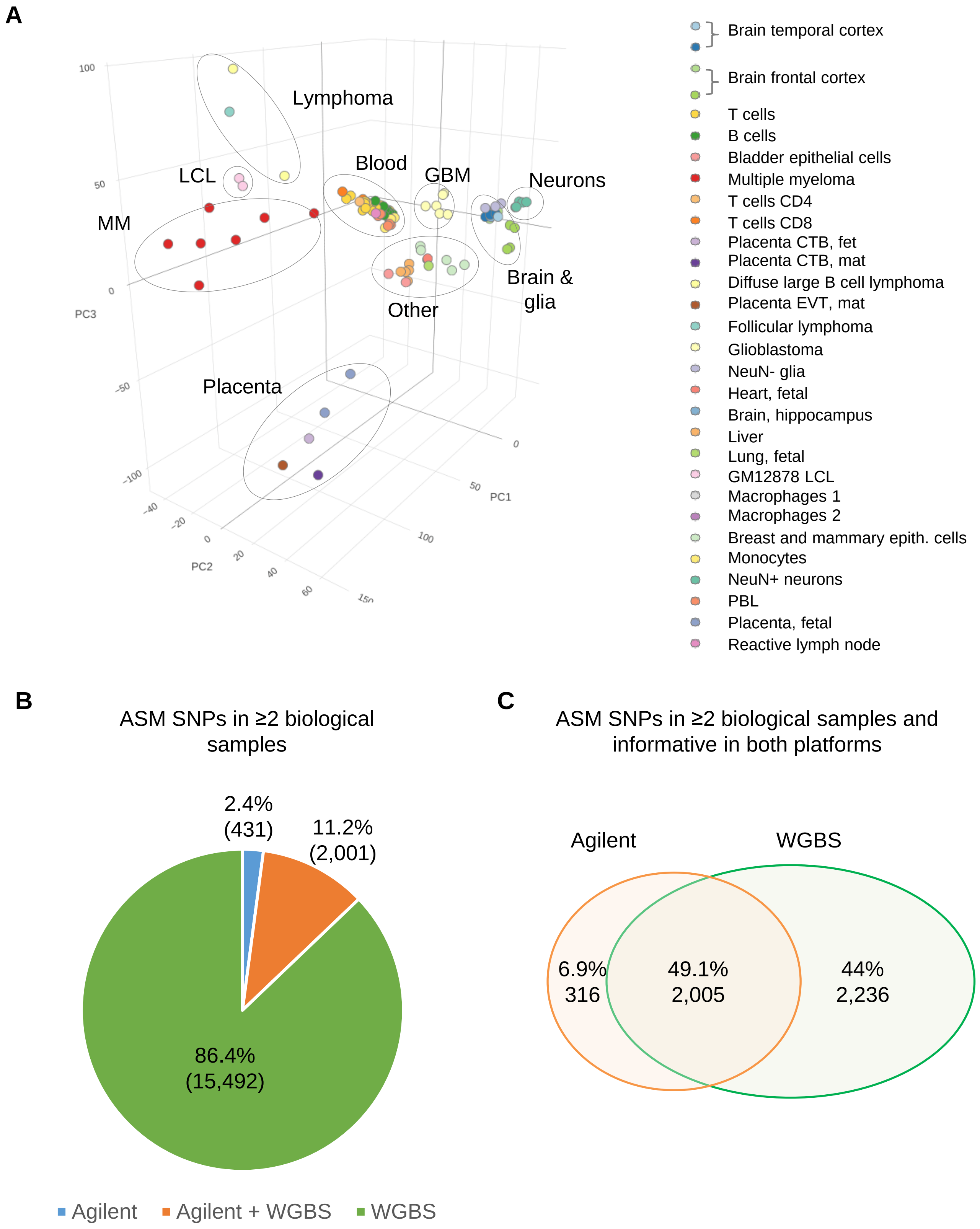
A
Lymphoma
LCL
MM
Blood
GBM
Neurons
Brain &
glia
Other
Placenta
PC1
PC2
PC3
Brain temporal cortex
Brain frontal cortex
T cells
B cells
Bladder epithelial cells
Multiple myeloma
T cells CD4
T cells CD8
Placenta CTB, fet
Placenta CTB, mat
Diffuse large B cell lymphoma
Placenta EVT, mat
Follicular lymphoma
Glioblastoma
NeuN- glia
Heart, fetal
Brain, hippocampus
Liver
Lung, fetal
GM12878 LCL
Macrophages 1
Macrophages 2
Breast and mammary epith. cells
Monocytes
NeuN+ neurons
PBL
Placenta, fetal
Reactive lymph node
B
ASM SNPs in ≥2 biological
samples
2.4%
(431)
11.2%
(2,001)
86.4%
(15,492)
Agilent
Agilent + WGBS
WGBS
C
ASM SNPs in ≥2 biological samples and
informative in both platforms
Agilent
WGBS
6.9%
316
49.1%
2,005
44%
2,236

Fig. S4. Distribution of ASM shows a high proportion of rare or private ASM in both cancer and normal samples and a significant increase in per-sample ASM in the cancers.

A, Most of the ASM calls are found only in one sample. While many might be genuine ASM associated with rare SNPs or with inter-individual variability, all downstream analyses in the current study are focused on recurrent ASM detected in at least two samples. **B** and **C**, Approximately one third of the ASM DMRs were identified only in cancer samples (referred to here as “cancer-only ASM”). Given that our study included more non-cancer than cancer samples, this high proportion of ASM SNPs found only in the cancers is significantly increased compared to random expectation.

Fig. S4

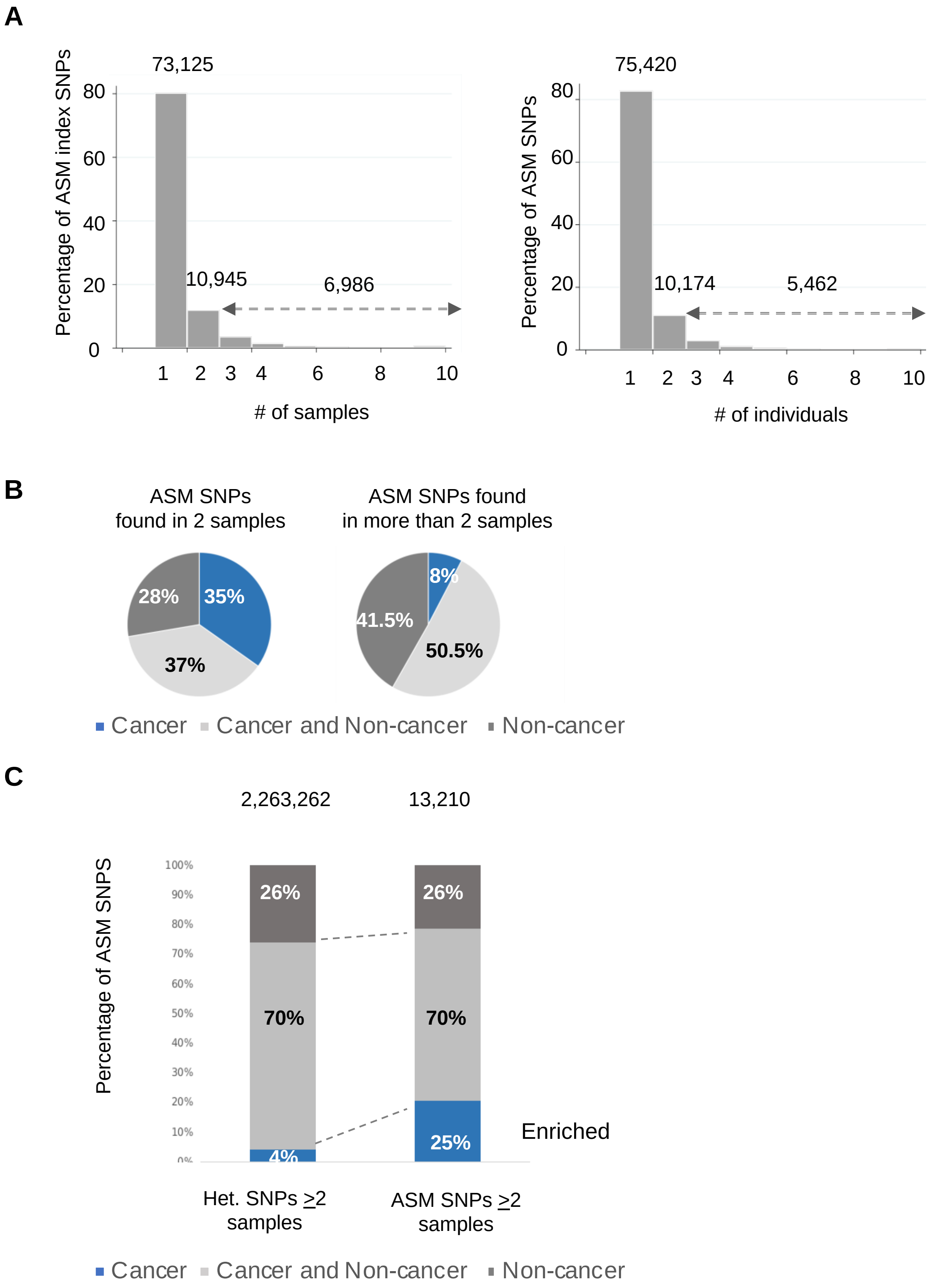
A
73,125
Percentage of ASM index SNPs
80
60
40
20
0
10,945
6,986
1 2 3 4 6 8 10
# of samples
75,420
Percentage of ASM SNPs
10,174
5,462
# of individuals
B
ASM SNPs found in 2 samples
ASM SNPs found in more than 2 samples
28%
35%
37%
8%
41.5%
50.5%
Cancer
Cancer and Non-cancer
Non-cancer
C
2,263,262
13,210
Percentage of ASM SNPS
26%
70%
4%
25%
Enriched
Het. SNPs ≥2 samples
ASM SNPs ≥2 samples
Cancer
Cancer and Non-cancer
Non-cancer

Fig. S5. Comparison of results from individual DNA samples analyzed by two WGBS library construction kits at two sequencing facilities, or by SureSelect and WGBS.

A, Cross platform and library correlations between net methylation showing a good R^2 coefficient ranging from 0.94 to 0.96 for the 9 samples assessed by two WGBS library preparation protocols or two platforms (SureSelect, WGBS), and improve in regions with deeper coverage. **B**, Cross platform and cross-library correlations between allelic methylation differences, showing that most of the ASM found in only one facility or one platform shows the same methylation difference trend (i.e. “direction” of the allelic methylation bias) in both facilities, but is sub-threshold in the data from one facility, in terms of p-value and/or number of ASM CpGs.

Fig. S5

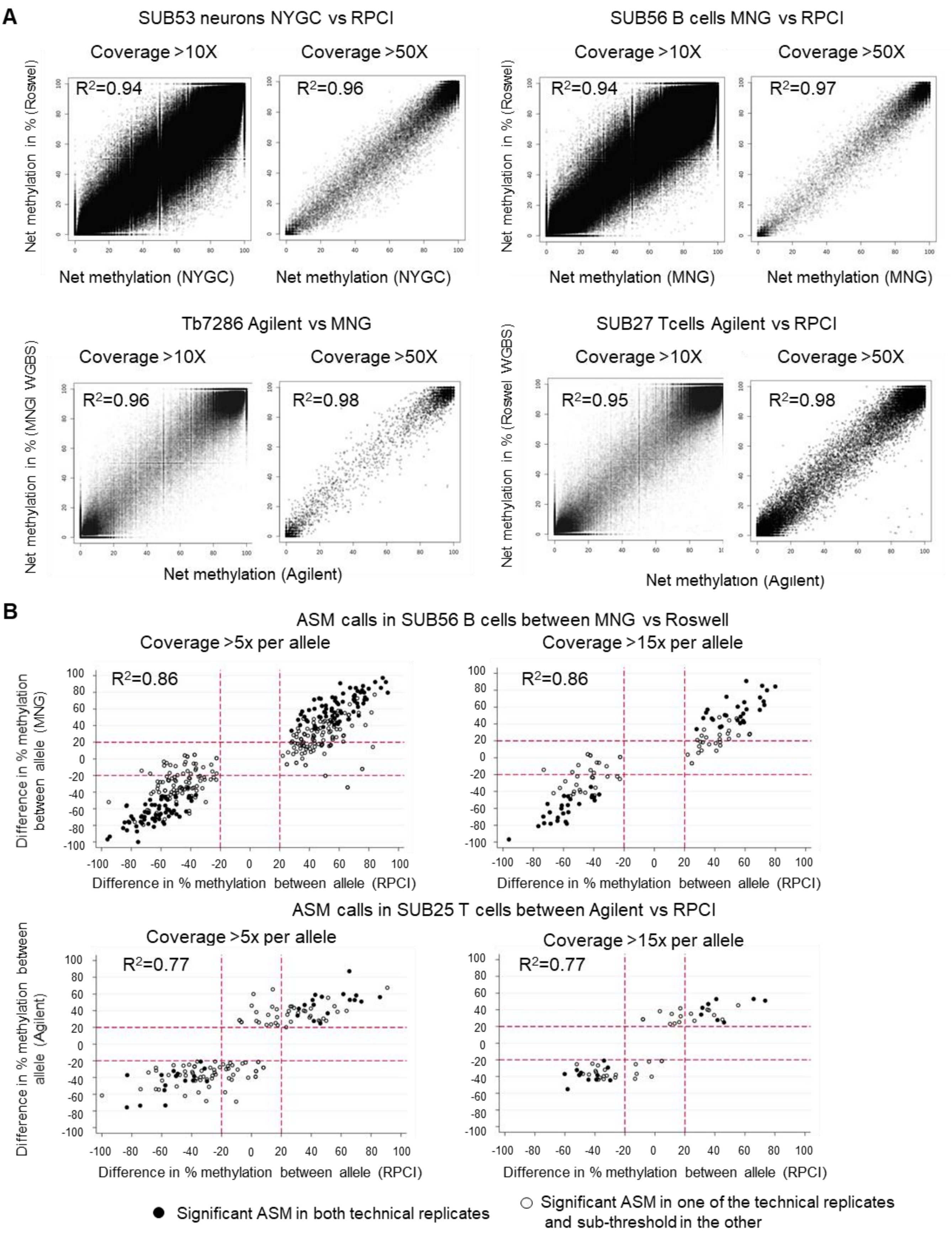
A
SUB53 neurons NYGC vs RPCI
Coverage >10X
Coverage >50X
R2=0.94
R2=0.96
Net methylation in % (Roswel)
Net methylation (NYGC)
SUB56 B cells MNG vs RPCI
Coverage >10X
Coverage >50X
R2=0.94
R2=0.97
Net methylation in % (Roswel)
Net methylation (MNG)
Tb7286 Agilent vs MNG
Coverage >10X
Coverage >50X
R2=0.96
R2=0.98
Net methylation in % (MNGI WGBS)
Net methylation (Agilent)
SUB27 Tcells Agilent vs RPCI
Coverage >10X
Coverage >50X
R2=0.95
R2=0.98
Net methylation in % (Roswel WGBS)
Net methylation (Agilent)
B
ASM calls in SUB56 B cells between MNG vs Roswell
Coverage >5x per allele
Coverage >15x per allele
R2=0.86
R2=0.86
Difference in % methylation between allele (MNG)
Difference in % methylation between allele (RPCI)
ASM calls in SUB25 T cells between Agilent vs RPCI
Coverage >5x per allele
Coverage >15x per allele
R2=0.77
R2=0.77
Difference in % methylation between allele (Agilent)
Difference in % methylation between allele (RPCI)
Significant ASM in both technical replicates
Significant ASM in one of the technical replicates and sub-threshold in the other

Fig. S6. Example of a chromosome region illustrating consistency between SureSelect methyl-seq, WGBS, and targeted bisulfite sequencing

A, Map of the region on chromosome 20 containing the ASM index SNP rs2427290. When covered in both SureSelect and WGBS, the net methylation is consistent between both assays, and shows low methylation "wells" at CpG islands, as expected. ASM dictated by index SNP rs2427290 is detected in both assays, with additional ASM SNPs found by WGBS, as expected. **B**, Primary sequencing data from WGBS, Agilent SureSelect methyl-seq, and targeted bis-seq, showing consistent findings of ASM in T cell samples. Rows represent sequence reads, and columns CpG sites in these reads. All samples are heterozygous, and the reads are separated by allele. Methylated CpGs are black circles, and unmethylated CpGs are white circles.

Fig. S6

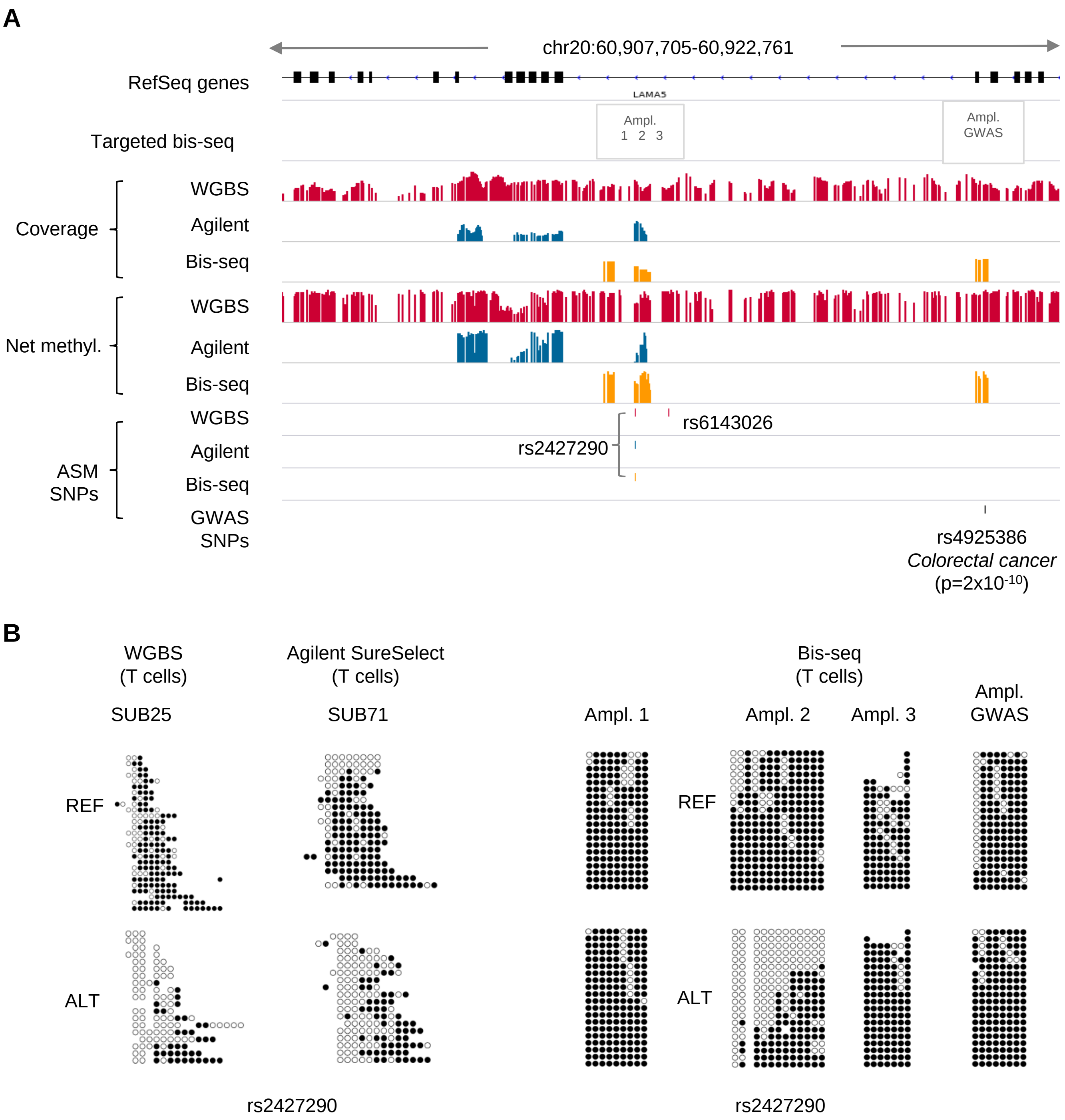

A
chr20:60,907,705-60,922,761
RefSeq genes
LAMA5
Targeted bis-seq
Ampl.
1 2 3
Ampl.
GWAS
Coverage
WGBS
Agilent
Bis-seq
Net methyl.
WGBS
Agilent
Bis-seq
ASM SNPs
WGBS
Agilent
Bis-seq
GWAS SNPs
rs6143026
rs2427290
rs4925386
Colorectal cancer
(p=2x10-10)
B
WGBS
(T cells)
SUB25
Agilent SureSelect
(T cells)
SUB71
Bis-seq
(T cells)
Ampl. 1
Ampl. 2
Ampl. 3
Ampl.
GWAS
REF
ALT
REF
ALT
rs2427290
rs2427290

Fig. S7. Validations of ASM DMRs in disease-associated chromosomal regions: rs10411630 and multiple sclerosis

A, Map showing the ASM region associated with rs10411630 in a putative tissue-specific promoter/enhancer region of the *CCDC155* gene, downstream of the *DKKL1* gene on chromosome 19. ENCODE chromatin state tracks suggest dynamic regulation, with active or quiescent marks depending on the cell type. Bisulfite PCR amplicons were designed to overlap the ASM and flanking SNPs, and to include at least 3 CpGs. The SNP is in high LD and R^2 (R^2 =0.98) with the rs2303759 GWAS peak SNP associated with multiple sclerosis, and another amplicon was designed to assess possible ASM at this position (which did not show ASM in our genome-wide data). The ASM index SNP disrupts a C-rich EGR1 TF binding motif and an EGR1 ChIP-seq peak found in K562 cells, supporting rs1041163 as a strong candidate rSNP. **B**, Targeted bis-seq reads, validating a discrete ASM regions (amplicon 2 and part of amplicon 3) spanning ~700 bp in T cells and brain. Number of ASM samples, informative tissue types and additional annotations are in **Additional file 3: Table S2**. Rows represent sequence reads, and columns CpG sites in these reads. All samples are heterozygous, and the reads are separated by allele. Methylated CpGs are black circles, and unmethylated CpGs are white circles.

Fig. S7

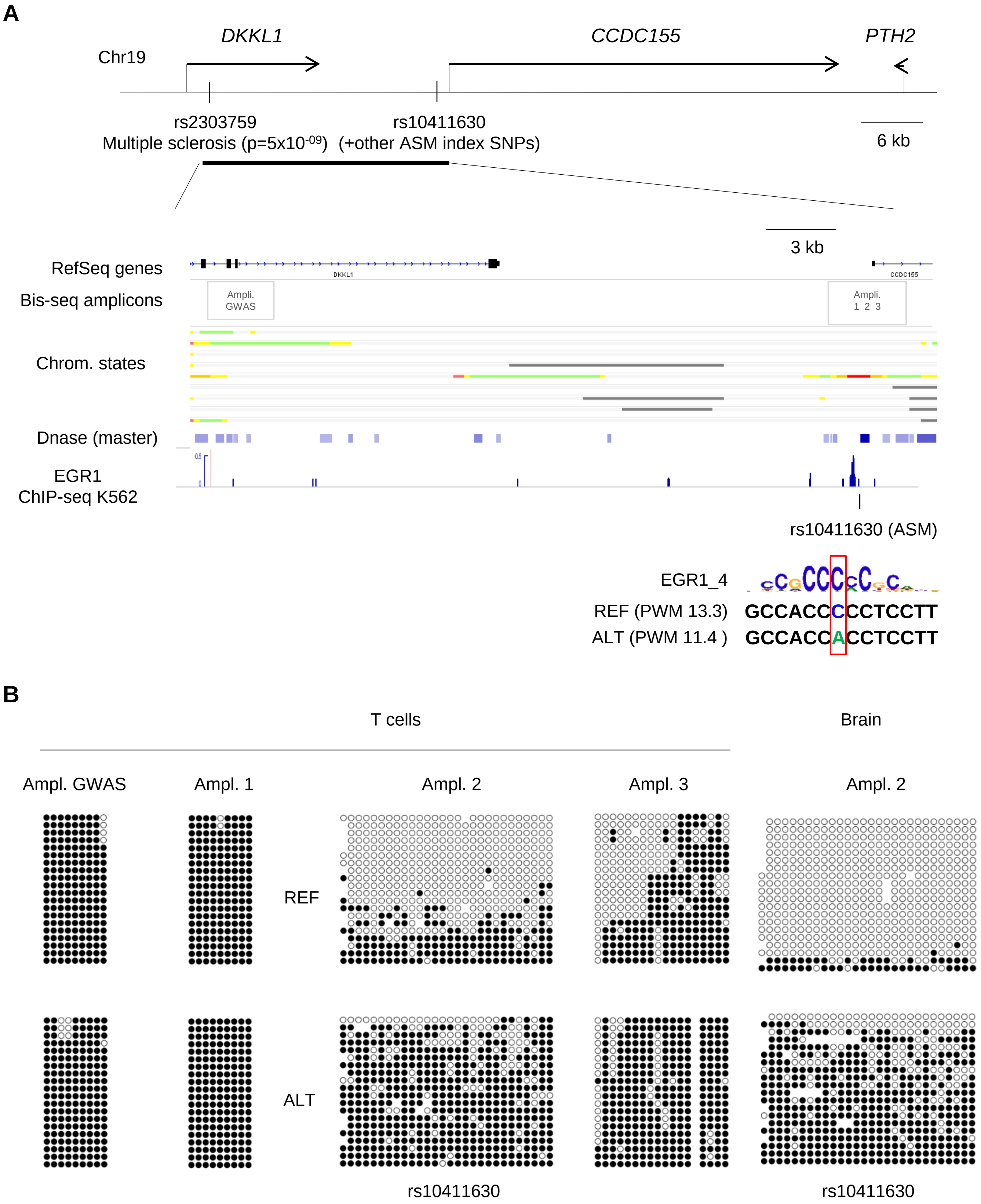
A
DKKL1
CCDC155
PTH2
Chr19
rs2303759
Multiple sclerosis (p=5x10-09)
rs10411630
(+other ASM index SNPs)
6 kb
3 kb
RefSeq genes
DKKL1
CCDC155
Bis-seq amplicons
Ampli. GWAS
Ampli. 1 2 3
Chrom. states
Dnase (master)
EGR1
ChIP-seq K562
0.5
0
rs10411630 (ASM)
EGR1_4
REF (PWM 13.3)
GCCACCCCCTCCTT
ALT (PWM 11.4)
GCCACCACCTCCTT
B
T cells
Brain
Ampl. GWAS
Ampl. 1
Ampl. 2
Ampl. 3
Ampl. 2
REF
ALT
rs10411630
rs10411630

Fig. S8. Validation of ASM DMRs in disease-associated chromosomal regions: rs2427290 and colorectal cancer

A, Map showing the ASM region tagged by SNP rs2427290 in the *LAMA5* gene on chromosome 20. The same region is shown, for a different purpose, in **Figure S5**. This region has an active promoter/enhancer state (color-coded red and yellow) in GM12878 (asterisk) but is in a Txn state (green) without promoter characteristics in other ENCODE cell lines. The ASM index SNP is close to (6.5 kb) and in moderate LD (lenient haplotype block; D'=0.8) with GWAS peak SNP rs4925386 associated with colorectal cancer. The ASM index SNP is in a region of open chromatin (DNAse hypersensitivity) and disrupts an ENCODE discovery motif for CCNT2 TF binding, located in a cluster of other TF binding sites and ENCODE ChIP-seq signals. The relatively hypomethylated allele is the one with the higher predicted CCNT2 binding affinity. For this occurrence, the motif maps to the negative strand and is reported from 3' to 5' as per atSNP convention. **B**, Targeted bis-seq reads validating a discrete ASM regions (amplicon 2) spanning ~400 bp in T cells and colonic mucosa. ASM is not found at the GWAS peak SNP. Numbers of ASM samples and additional annotations, are in **Additional file 3: Table S2**. Rows represent sequence reads, and columns CpG sites in these reads. All samples are heterozygous, and the reads are separated by allele. Methylated CpGs are black circles, and unmethylated CpGs are white circles.

Fig. S8

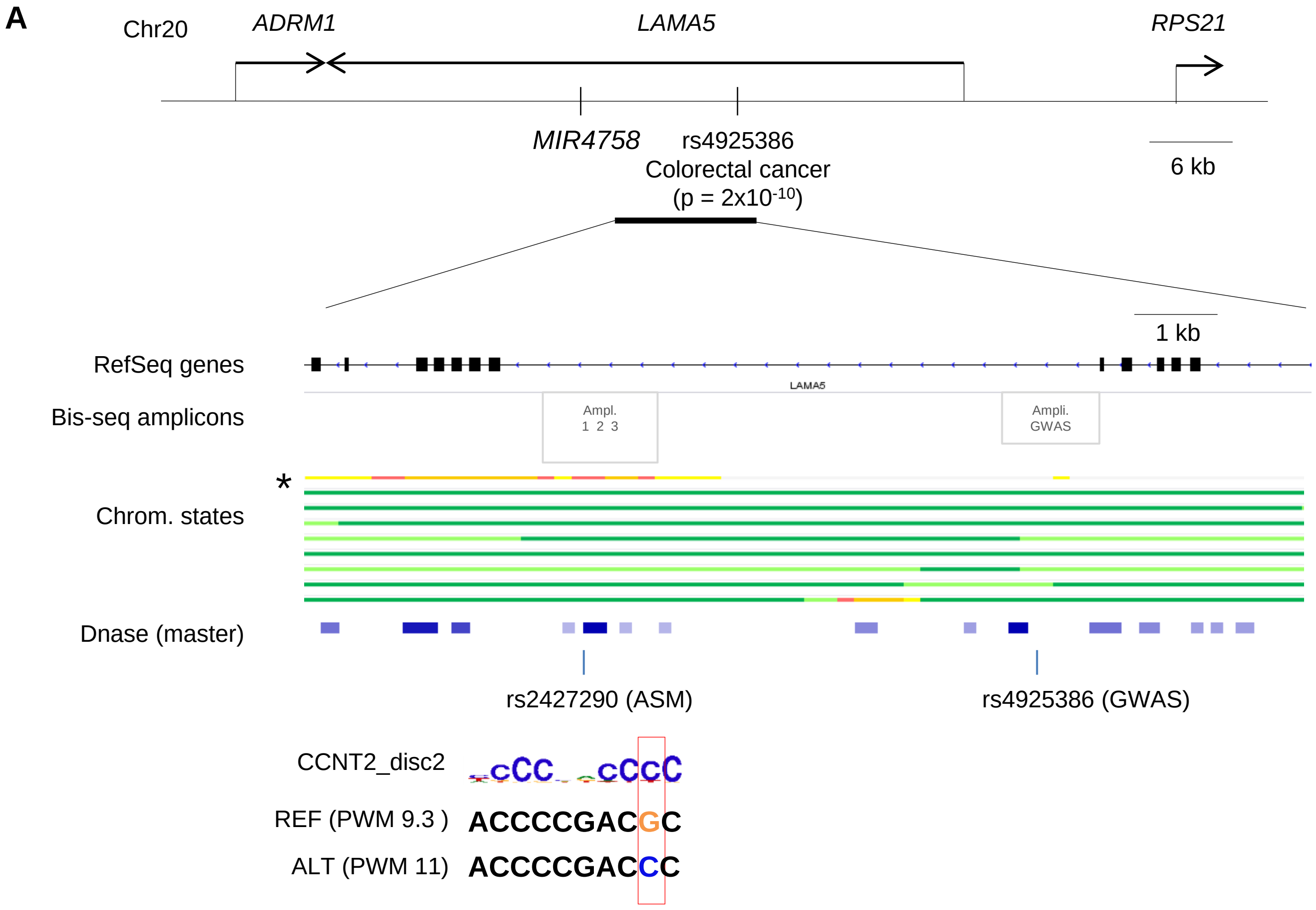
A
Chr20
ADRM1
LAMA5
RPS21
MIR4758
rs4925386
Colorectal cancer
(p = 2x10-10)
6 kb
1 kb
RefSeq genes
LAMA5
Bis-seq amplicons
Ampl.
1 2 3
Ampli.
GWAS
*
Chrom. states
Dnase (master)
rs2427290 (ASM)
rs4925386 (GWAS)
CCNT2_disc2
REF (PWM 9.3)
ACCCCGACGC
ALT (PWM 11)
ACCCCGACCC

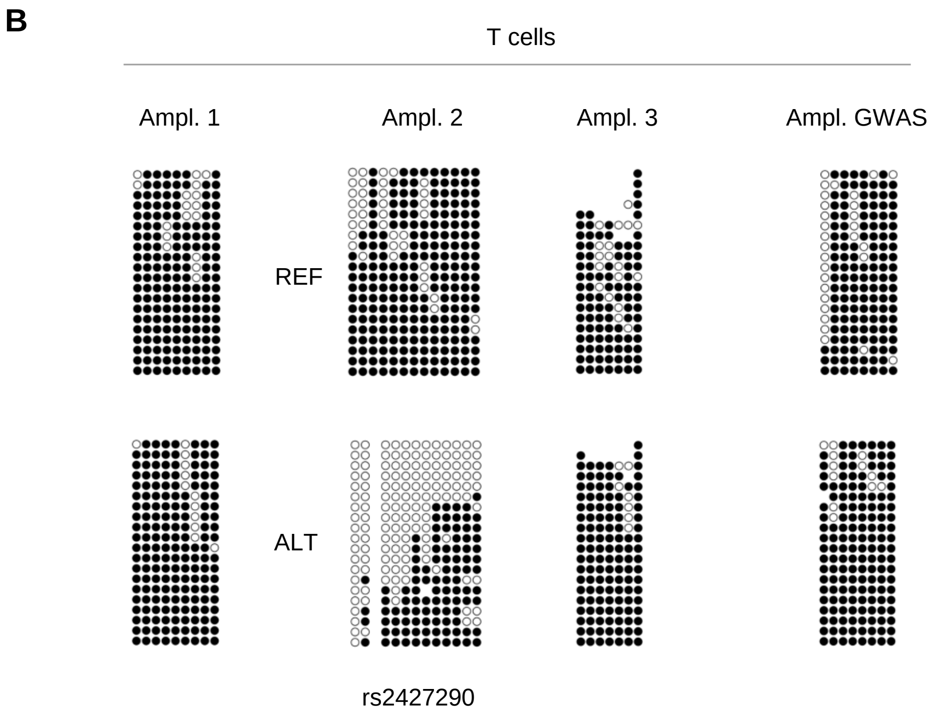
B
T cells
Ampl. 1
Ampl. 2
Ampl. 3
Ampl. GWAS
REF
ALT
rs2427290

Fig. S9. Validation of ASM DMRs in disease-associated chromosomal regions: rs2283639 and non-small cell lung carcinoma

A, Map showing the ASM region tagged by index SNP rs2283639, located in a promoter/enhancer region (color-code yellow and red) of the *ETS2* gene on chromosome 21. The SNP is very close to (4 kb distance) and in partial LD (lenient haplotype block; D'=.96) with GWAS peak SNP rs1209950, associated with survival after treatment of non-small cell lung carcinoma. The ASM index SNP disrupts an ENCODE-discovery motif for SMC3 (cohesion complex component), and it co-localizes with a CTCF ChIP-seq peak and a and weak SMC3 ChIP-seq peak, in a cluster of multiple TF binding sites and ENCODE ChIP-seq signals. There is also disruption of an ETS1 motif, which could play a role as negative or positive feedback on *ETS2* gene expression. For this occurrence, these two motifs map to the negative strand and are reported from 3' to 5'. Three amplicons were designed for targeted bis-seq of the ASM region. **B**, Graphical representation of the targeted bis-seq results, validating a discrete ASM regions (amplicon 2) spanning ~600 bp in T cells and lung. The relatively hypomethylated allele is the one with higher predicted SMC3 and ETS1_2 binding affinity. Numbers of ASM samples in each tissue and cell type, and additional annotations, are in **Additional file 3: Table S2**. Rows represent sequence reads, and columns CpG sites in these reads. All samples are heterozygous, and the reads are separated by allele. Methylated CpGs are black circles, and unmethylated CpGs are white circles.

Fig. S9

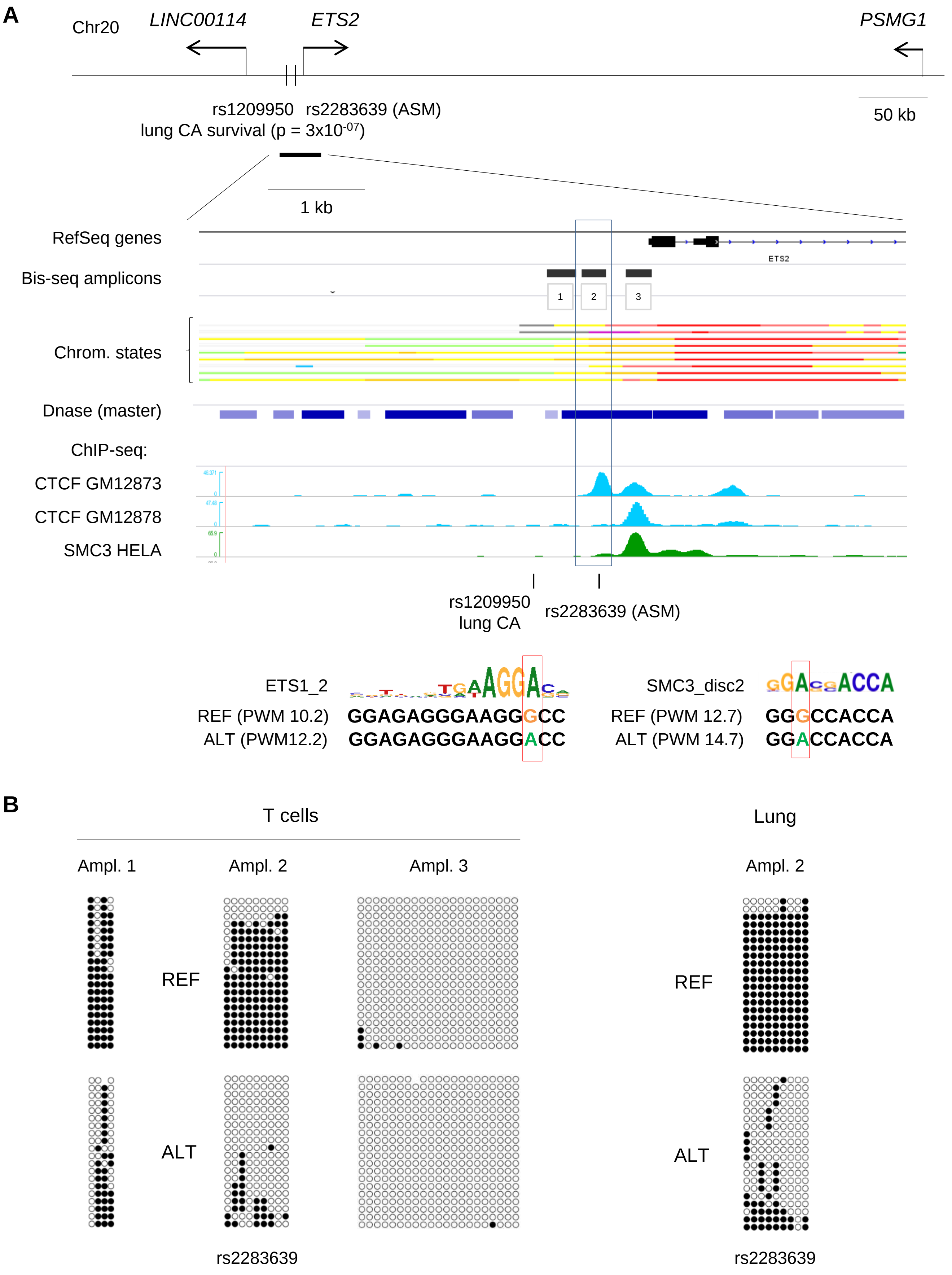
A
Chr20
LINC00114
ETS2
PSMG1
rs1209950
rs2283639 (ASM)
lung CA survival (p = 3x10-07)
50 kb
1 kb
RefSeq genes
ETS2
Bis-seq amplicons
1
2
3
Chrom. states
Dnase (master)
ChIP-seq:
CTCF GM12873
CTCF GM12878
SMC3 HELA
rs1209950
lung CA
rs2283639 (ASM)
ETS1_2
REF (PWM 10.2) GGAGAGGGAAGGGCC
ALT (PWM12.2) GGAGAGGGAAGGACC
SMC3_disc2
REF (PWM 12.7) GGGCCACCA
ALT (PWM 14.7) GGACCACCA
B
T cells
Lung
Ampl. 1
Ampl. 2
Ampl. 3
Ampl. 2
REF
ALT
REF
ALT
rs2283639
rs2283639

Fig. S10. Validations of ASM DMRs spanning a range of ASM ranks

Targeted bis-seq showing validation of additional ASM regions (others in **Figures S7-S9**), with ASM index SNPs that have high, middle or low overall ranks. The results of all validations (27 ASM DMRs tested) are summarized in **Table S6**. Samples are heterozygous, and the reads are separated by allele. Methylated CpGs are black circles, and unmethylated CpGs are white circles. In each illustrated case, the relatively hypermethylated allele (REF or ALT) in the targeted bis-seq data is consistent with the relatively hypermethylated allele detected in the primary SureSelect or WGBS data (**Additional file 3: Table S2**). Note that since the calculation of overall rank incorporates both the ASM strength and the percentage of heterozygotes showing ASM, rare individuals can show strong ASM even in loci with weak (numerically high) overall ranks. An example is the strong ASM tagged by index SNP rs35907548 in the illustrated T cell sample.

Fig. S10

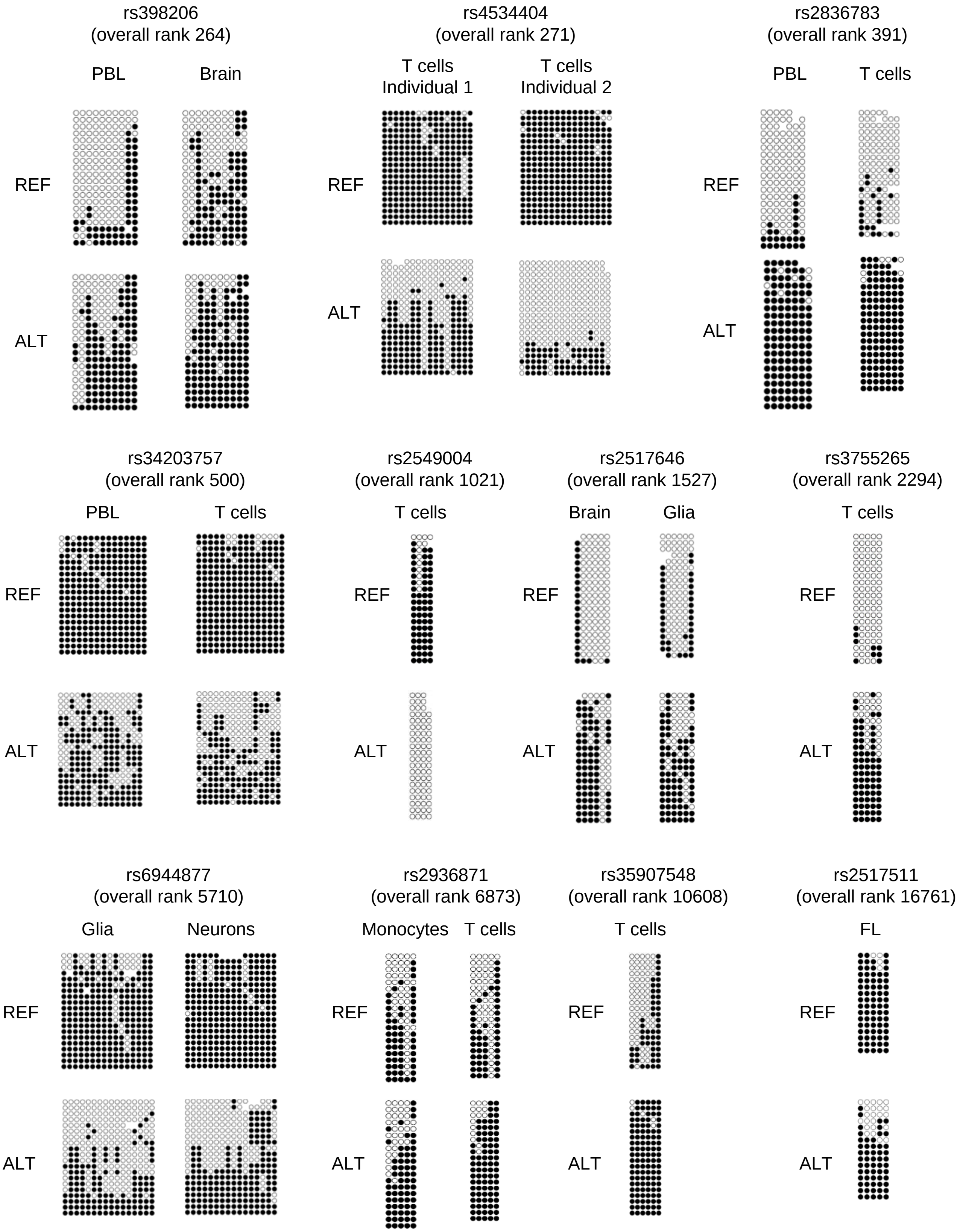

Complete list of tested loci is in Table S6

Fig. S11. Kernel density plots of methylation levels showing global hypomethylation and decrease in the percentage of highly methylated CpGs in cancers

Distribution of the averaged percentage of net methylation genome wide for all informative CpGs in cancer and lineage matched normal samples, by Kernel density estimation. As expected, CpG methylation has a bimodal distribution with a large major mode of high methylated CpGs (>80%) and a small minor mode of low methylated CpGs (<5% methylation; corresponding to CpG-islands) in the non-neoplastic cell type (B cells and glia). In multiple myeloma and lymphoma, prominent global hypomethylation with the loss of the high methylated CpGs peak is observed. Global hypomethylation is present, but milder, in GBMs.

Fig. S11

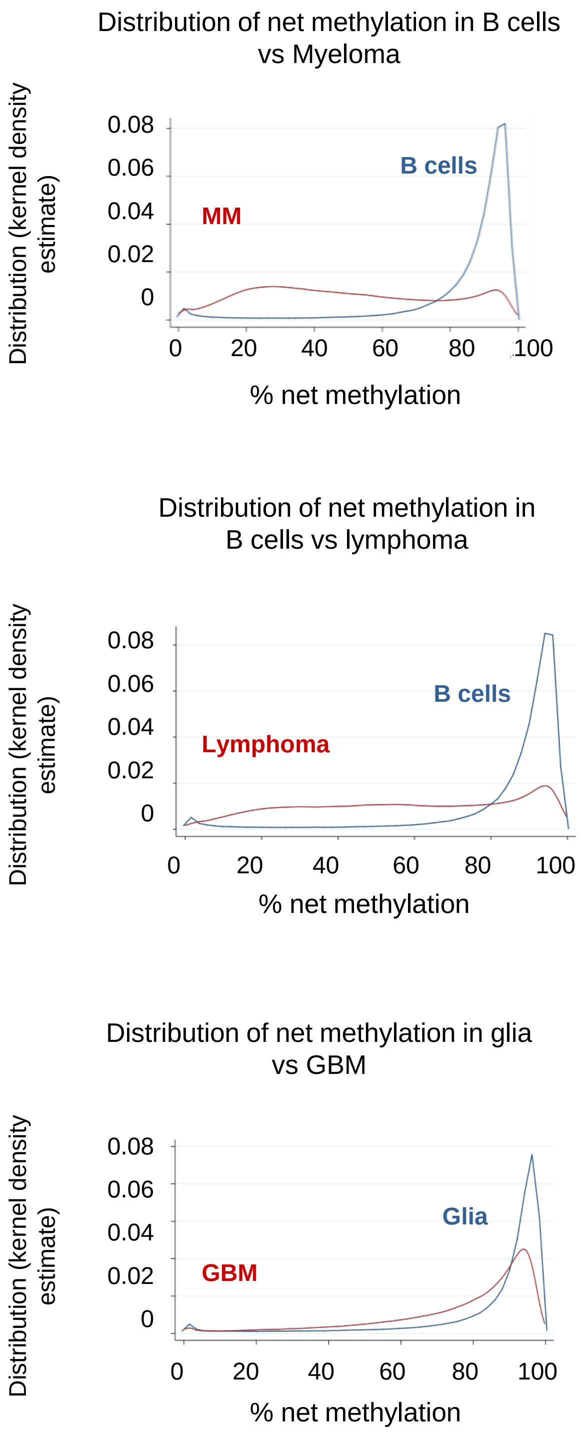
Distribution of net methylation in B cells vs Myeloma
Distribution (kernel density estimate)
0.08
0.06
0.04
0.02
0
B cells
MM
0
20
40
60
80
100
% net methylation
Distribution of net methylation in B cells vs lymphoma
Distribution (kernel density estimate)
0.08
0.06
0.04
0.02
0
B cells
Lymphoma
0
20
40
60
80
100
% net methylation
Distribution of net methylation in glia vs GBM
Distribution (kernel density estimate)
0.08
0.06
0.04
0.02
0
Glia
GBM
0
20
40
60
80
100
% net methylation

Fig. S12. Replication of the findings using WGBS from a single facility

Gains of ASM are strongly correlated with genome-wide DNA hypomethylation, and ASM correlates with allele-specific binding affinities of specific classes of CTCF and TF recognition motifs in both cancer and normal samples, using WGBS data from a single sequencing facility (RPCI), which utilized a single library construction method. **A,** Data from WGBS performed at RPCI, showing the relationship between global DNA methylation and the percentage of informative SNPs that reveal ASM in each sample. The results confirm a strong inverse correlation between per sample ASM frequencies and global methylation levels. Cancer samples (color-coded in red scale) have higher ASM frequencies than nearly all the non-cancer samples (blue scale), except for overlap of the GBM data points with normal placental trophoblast and cultured bladder epithelial cells. When compared to lineage-matched normal cell types, there is a 7 to 12-fold increase in ASM frequencies in the three types of cancers. The EBV-immortalized GM12878 LCL shows global hypomethylation and a high frequency of ASM. **B,** Significant correlations between allelic TF binding affinity scores and ASM in each of the 4 classes of ASM loci, using data from the RPCI core facility. Left panel: fitted ASM difference on PWM score using a multivariate mixed model. The fitted line and its 95-confidence intervals (area) are shown for each ASM class; slopes were calculated by the marginal effects of the interaction term between PWM score and ASM class and were significantly different from zero. Correlations are similar in cancer ASM (in both non-desert and desert) compared to non-cancer ASM, with small differences in the slopes for each class. Right panel: pairwise comparisons of the correlations in each of the 4 classes of ASM loci, Bonferroni-adjusted for multiple testing. While all the slopes are in a similar range, the correlations in the mixed model are weakest for cancer-only ASM loci, with a modest but statistically significant difference between the cancer vs non-cancer ASM classes, but not between desert and non-desert ASM loci. N: number of occurrences included in the mixed model. **C,** X-Y plots showing examples of TF motifs with strong correlations between predicted allele-specific binding site affinities (estimated by PWM scores) and methylation differences across all occurrences showing ASM, using data from the RPCI facility. Each data point represents one occurrence of the motif overlapping an ASM index SNP in cancer (orange) or non-cancer samples (blue). For occurrences showing ASM in multiple samples, allelic methylation differences were averaged across samples by sample type. R^2 and B-H corrected p-values (FDR) were calculated using linear regression.

Fig. S12: results based on data from a single sequencing facility (RPCI), using a single library construction method.

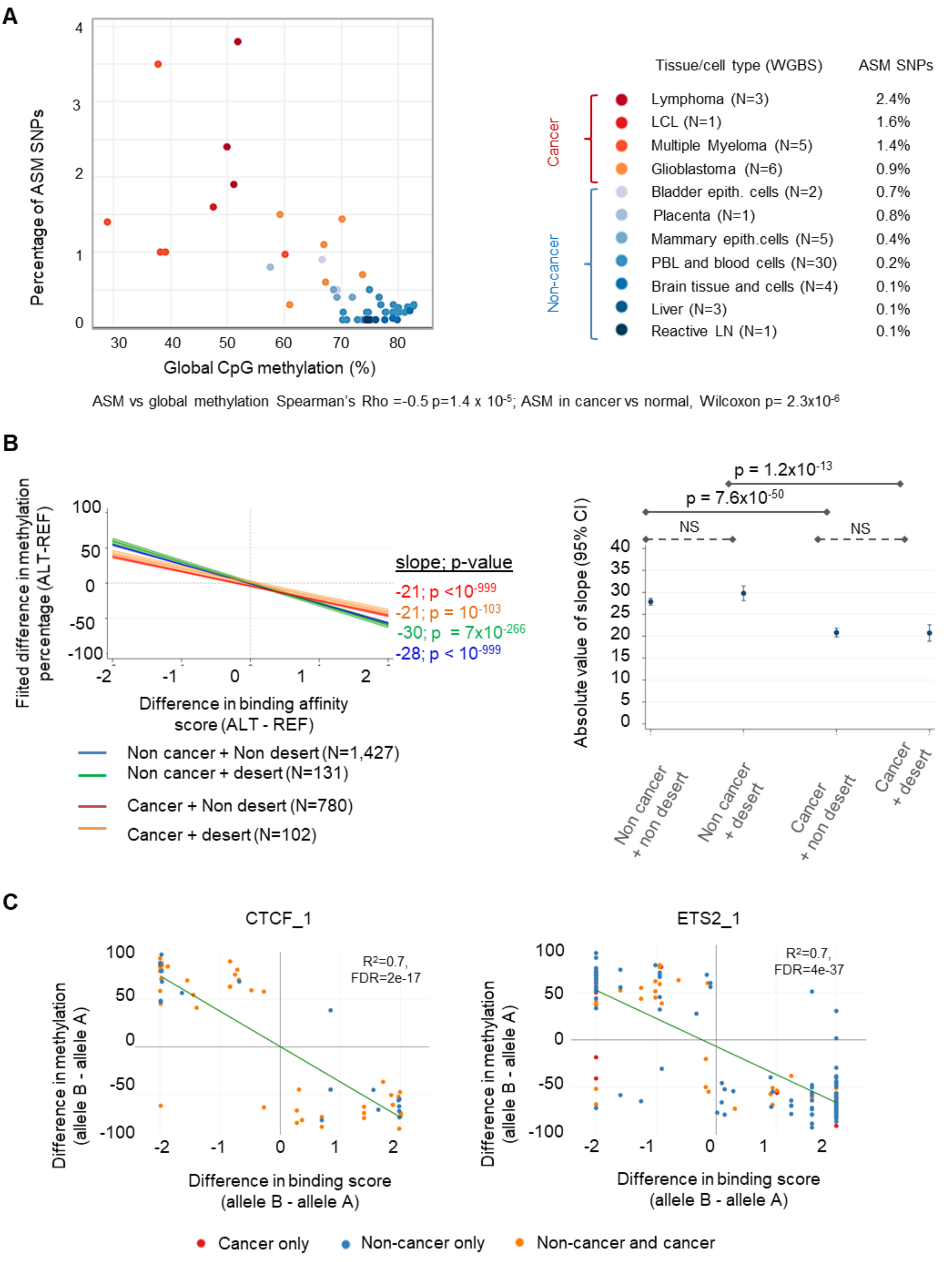

Fig. S13. Allele-specific losses of methylation leading to ASM in cancers

Graphs showing the fitted values of the percentage methylation in cancer (red) for myeloma, lymphoma and glioblastoma versus the lineage-matched non-neoplastic cell types (B cells for myeloma and lymphoma and glia for glioblastoma) for regions where ASM was found only in cancer. In non-neoplastic cells, on average, the methylation levels in these regions were high or intermediate on both alleles and ASM in cancer reflects losses of methylation on one of the alleles. The average fractional methylation was estimated using a linear mixed model with random intercept and random slope (Methods). The light lines represent the fitted values for each locus and the bold line the average fit. The slope between low and high methylation estimates the ASM magnitude. The non-significant and small slope in non-neoplastic cells reflects the absence of significant ASM in these regions.

Fig. S13

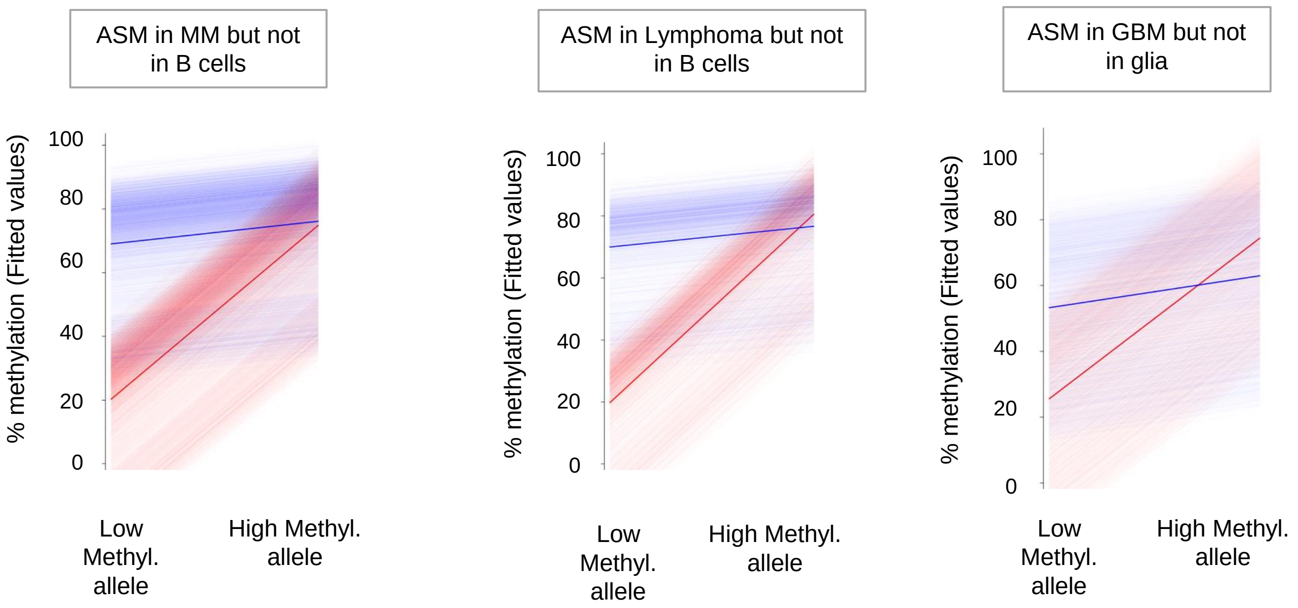
ASM in MM but not in B cells
ASM in Lymphoma but not in B cells
ASM in GBM but not in glia
% methylation (Fitted values)
100
80
60
40
20
0
Low Methyl. allele
High Methyl. allele

Fig. S14. Kernel density plots of methylation level distributions showing statistically enriched instances of allele-specific gains of methylation in cancers.

These Kernel density plots show the distribution of methylation values in non-neoplastic cells comparing methylation at loci where ASM was found neither in the non-neoplastic cells nor in the matched cancer samples vs loci where ASM was found only in the matched cancer. These graphs show that allele-specific loss of methylation (LOM), which represents the most common scenario for cancer-only ASM, is numerically predominant but is nonetheless relatively under-represented compared to random expectation in the globally hypomethylated genomic background, while the less frequent allelic-specific gains of methylation (GOM) are over-represented relative to this background. As shown in **Figure 3**, these instances of GOM in the cancers often map to regions of poised chromatin.

Fig. S14

Distribution of net methylation in B cells - specifically for loci that show ASM in myeloma (BLUE) vs loci without ASM (RED)

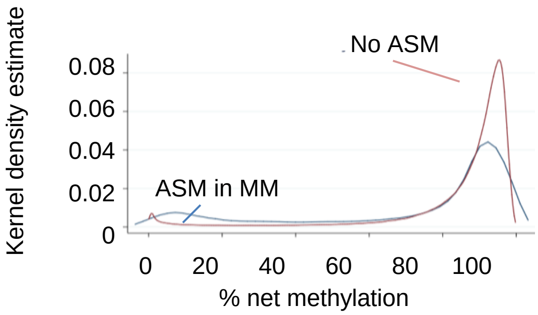

Over-representation of low methylation (OR: 4.1, $p< 10^{-999}$)

Under-representation of high methylation (OR: 0.3, $p=6x10^{-290}$)

Distribution of net methylation in B cells - specifically for loci that show ASM in lymphoma (BLUE) vs loci without ASM (RED)

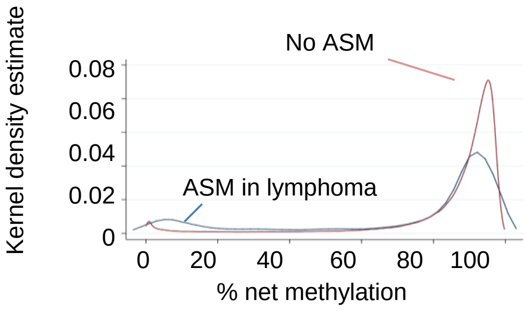

Over-representation of low methylation (OR: 3.9, $p=2x10^{-146}$)

Under-representation of high methylation (OR: 0.3, $p= 10^{-133}$)

Distribution of net methylation in glia - specifically for loci that show ASM in GBM (BLUE) vs loci without ASM (RED)

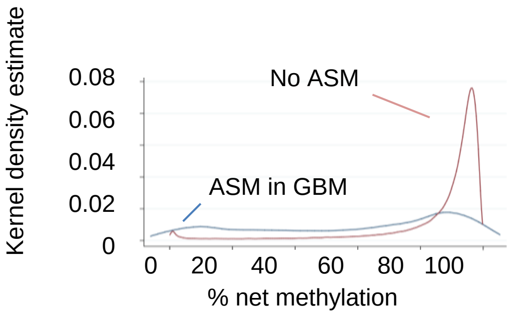

Over-representation of low methylation (OR: 6.9, $p<10^{-999}$)

Under-representation of high methylation (OR: 0.1, $p<10^{-999}$)

Fig. S15. Shared ASM loci in cancer and non-cancer have similar ASM magnitude

Graphs and diagrams showing the fitted values of the average percent methylation of the low and high methylated alleles in cancer (RED) for multiple myeloma (MM), lymphoma, and glioblastoma multiforme (GBM) vs cell lineage-matched non-neoplastic cell types (BLUE), namely B cells for MM and lymphoma and glia for GBM, for DMRs where ASM was found both in cancer and non-cancer. The average fractional methylation of each allele and in each cancer or normal sample class (middle panels) was estimated using a linear mixed model with random intercept and random slope (Methods).On the left panels, the light lines represent the fitted values for each locus and the bold line the average fit. The slopes between low and high methylated alleles estimate the ASM magnitude and are similar (parallel) in cancer and non-cancer samples, with a non-significant statistical interaction between cancer vs normal status and ASM magnitude. The right panels show primary WGBS data for representative examples, with sequence reads separated by allele. Methylated CpGs are black circles, and unmethylated CpGs are white circles.

Fig. S15

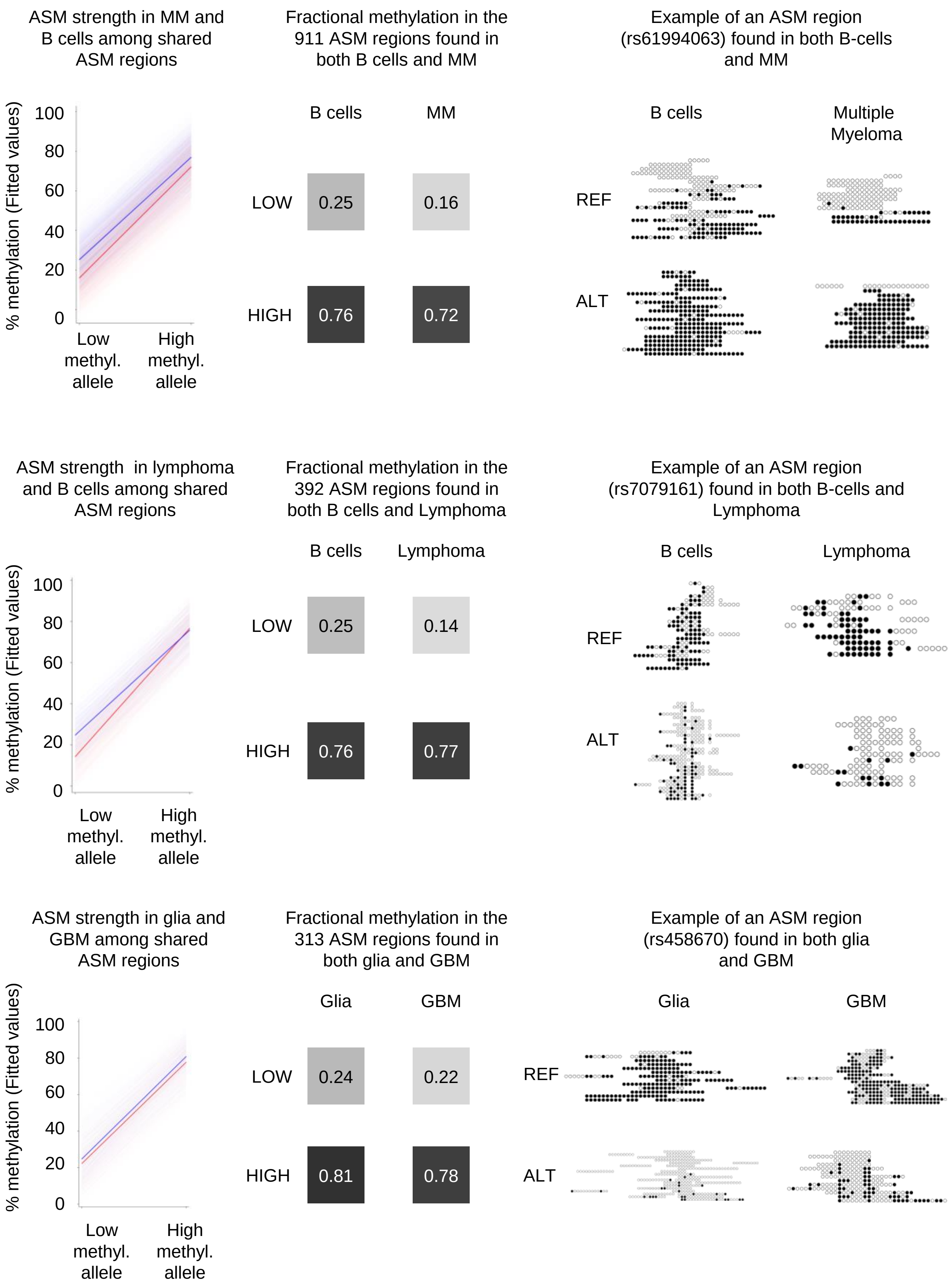
ASM strength in MM and B cells among shared ASM regions
% methylation (Fitted values)
100
80
60
40
20
0
Low methyl. allele
High methyl. allele
Fractional methylation in the 911 ASM regions found in both B cells and MM
B cells
MM
LOW
0.25
0.16
HIGH
0.76
0.72
Example of an ASM region (rs61994063) found in both B-cells and MM
B cells
Multiple Myeloma
REF
ALT
ASM strength in lymphoma and B cells among shared ASM regions
% methylation (Fitted values)
100
80
60
40
20
0
Low methyl. allele
High methyl. allele
Fractional methylation in the 392 ASM regions found in both B cells and Lymphoma
B cells
Lymphoma
LOW
0.25
0.14
HIGH
0.76
0.77
Example of an ASM region (rs7079161) found in both B-cells and Lymphoma
B cells
Lymphoma
REF
ALT
ASM strength in glia and GBM among shared ASM regions
% methylation (Fitted values)
100
80
60
40
20
0
Low methyl. allele
High methyl. allele
Fractional methylation in the 313 ASM regions found in both glia and GBM
Glia
GBM
LOW
0.24
0.22
HIGH
0.81
0.78
Example of an ASM region (rs458670) found in both glia and GBM
Glia
GBM
REF
ALT

Fig. S16. Correlations between allelic TF binding affinity scores and ASM magnitude in the 4 classes of ASM loci

A, Pairwise comparisons of the correlations in each of the 4 classes of ASM loci, Bonferroni-adjusted for multiple testing. While all the slopes are in a similar range, the correlations in the mixed model are weakest for cancer-only ASM loci, with a modest but statistically significant difference between the cancer vs non-cancer ASM classes, but not between desert and non-desert ASM loci. N: number of occurrences included in the mixed model. **B,** Graphs showing significant correlations between allelic TF binding affinity scores and ASM in the indicated two classes of ASM loci. The left panel shows the fitted ASM difference on PWM score using a multivariate mixed model. The fitted line and its 95-confidence intervals (area) are shown for each ASM class. The slopes of the fitted lines were calculated by the marginal effects of the interaction term between PWM score and ASM class and were significantly different from zero. The correlations are similar in cancer ASM compared to non-cancer ASM, with slightly weaker slope in cancer. The right panel shows the pairwise comparison of the correlations in the indicated two classes of ASM loci with a significant difference between the cancer vs non-cancer ASM classes. N: number of occurrences included in the mixed model. **C**, These examples were selected requiring at least 3 occurrences per ASM classes. The X-Y plots show ASM magnitude vs differences in predicted allele-specific binding affinities (PWM scores) for the EHF_1, SPI1_3, SPIB_2 and ETV6_1 motifs. All 4 classes of ASM loci show similar anti-correlations, but there is a slight decrease in the slope for cancer compared to normal ASM. Desert vs non-desert classes of ASM loci show essentially identical slopes. Regression lines were not plotted if there were less than 3 occurrences within the ASM class (non-cancer/desert for SPIB_2 and cancer/desert for ETV6_1).

Fig. S16

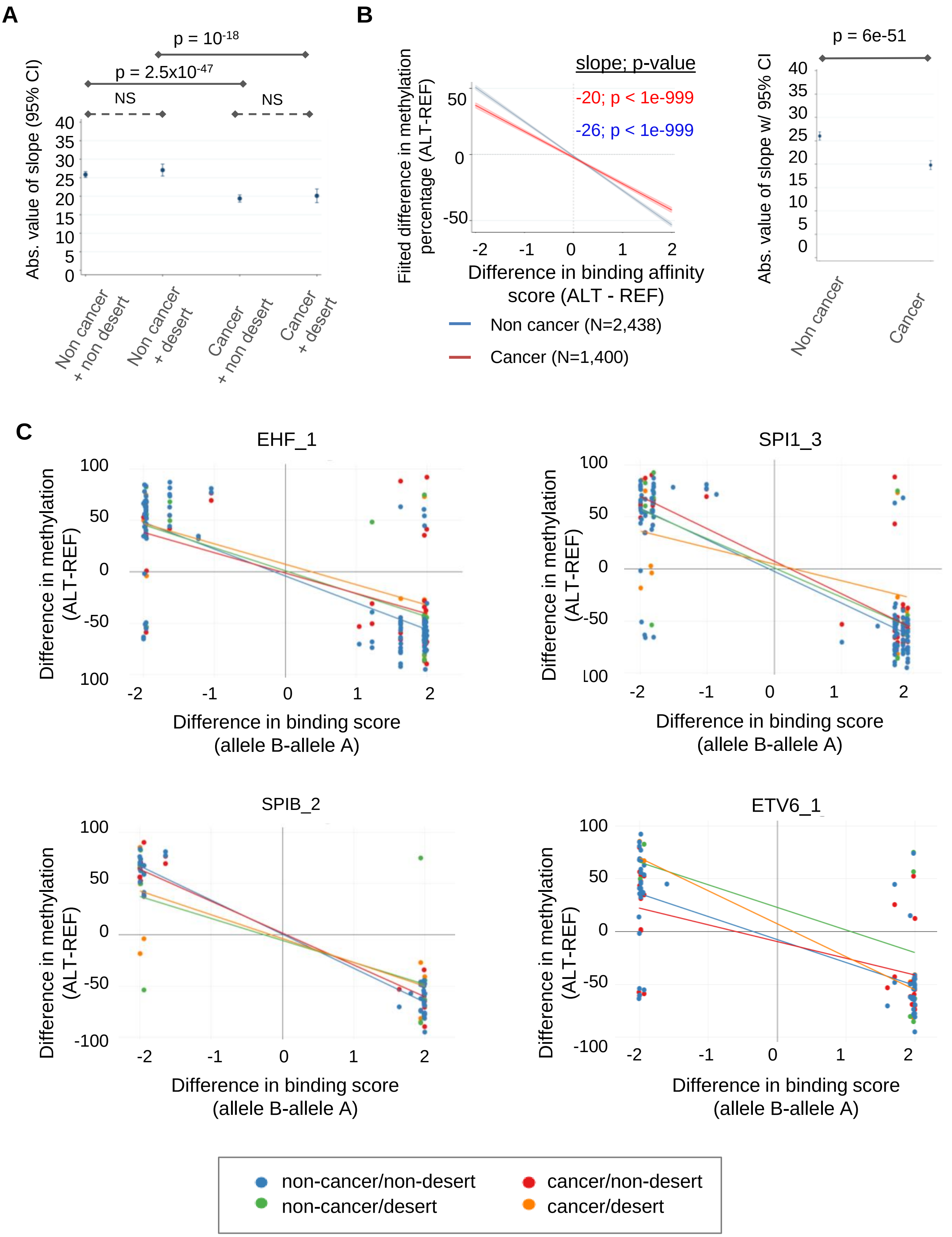
A
p = 10-18
p = 2.5x10-47
NS
NS
Abs. value of slope (95% CI)
40
35
30
25
20
15
10
5
0
Non cancer + non desert
Non cancer + desert
Cancer + non desert
Cancer + desert
B
slope; p-value
-20; p < 1e-999
-26; p < 1e-999
Fited difference in methylation percentage (ALT-REF)
50
0
-50
-2
-1
0
1
2
Difference in binding affinity score (ALT - REF)
Non cancer (N=2,438)
Cancer (N=1,400)
p = 6e-51
Abs. value of slope w/ 95% CI
Non cancer
Cancer
C
EHF_1
SPI1_3
SPIB_2
ETV6_1
Difference in methylation (ALT-REF)
100
50
0
-50
-100
Difference in binding score (allele B-allele A)
non-cancer/non-desert
non-cancer/desert
cancer/non-desert
cancer/desert

Fig. S17. Examples of ASM DMRs in chromatin deserts

A, Map showing the ASM DMR tagged by index SNP rs2272697 in the body of the *MANBA* gene. ENCODE chromatin state data show that this region is marked only by a non-regulatory chromatin state (Txn, color-coded green), and the index SNP has a weak regulomeDB score (5), this SNP in fact disrupts multiple ETS-family binding motifs, suggesting that it could have a regulatory role via an ETS-family transcriptional pathway at some stage of cellular differentiation. This SNP is in high LD (R2>0.9) with multiple GWAS peak SNPs (rs5026472, rs1054037 and rs7665090) associated lymphocyte count, liver cirrhosis, and multiple sclerosis. **B,** Map showing the ASM DMR tagged by index SNP rs13097644 in the intergenic region upstream of the *SETMAR* gene. This region is flagged as quiescent by ENCODE chromatin state (color-coded light gray), consistent with the low regulomeDB score (6) for this SNP. However, the index ASM SNP disrupts an ASM-correlated Erg TF binding motif, suggesting that rs13097644 might act as a regulatory genetic variant at some stage of cell differentiation.

Fig. S17

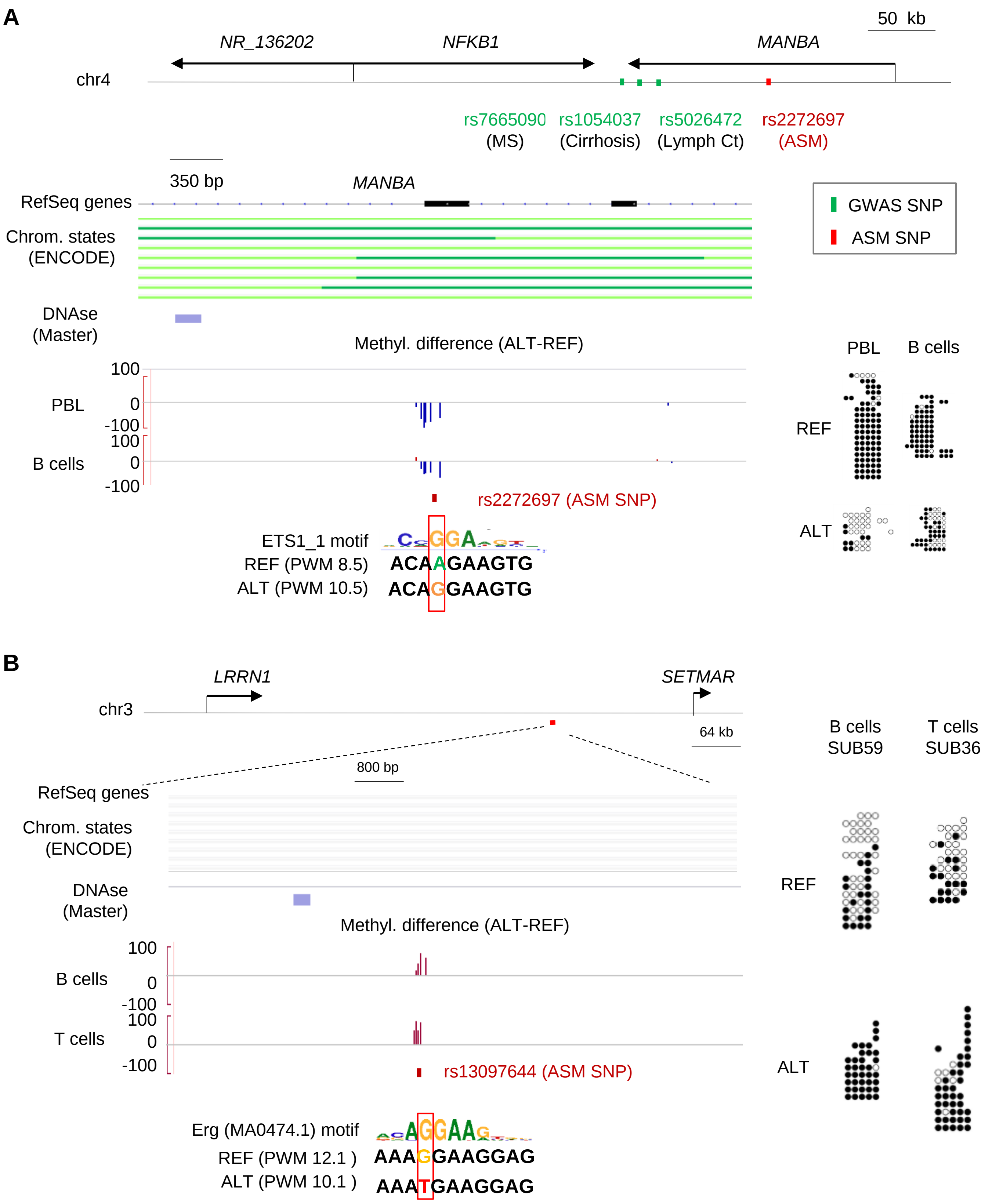
A
50 kb
NR_136202
NFKB1
MANBA
chr4
rs7665090 (MS)
rs1054037 (Cirrhosis)
rs5026472 (Lymph Ct)
rs2272697 (ASM)
350 bp
MANBA
RefSeq genes
Chrom. states (ENCODE)
GWAS SNP
ASM SNP
DNAse (Master)
Methyl. difference (ALT-REF)
PBL
B cells
100
0
-100
rs2272697 (ASM SNP)
ETS1_1 motif
REF (PWM 8.5) ACAAGAAGTG
ALT (PWM 10.5) ACAGGAAGTG
REF
ALT
B
LRRN1
SETMAR
chr3
64 kb
800 bp
RefSeq genes
Chrom. states (ENCODE)
DNAse (Master)
Methyl. difference (ALT-REF)
B cells
T cells
rs13097644 (ASM SNP)
Erg (MA0474.1) motif
REF (PWM 12.1) AAAGGAAGGAG
ALT (PWM 10.1) AAATGAAGGAG
B cells SUB59
T cells SUB36

Fig. S18. Models for inter-individual variability and allele-switching at ASM loci

A, ASM is not present, with high methylation on both alleles, when either the TFBS is not accessible (closed chromatin) or the TF is not sufficiently expressed (left panel). For accessible TFBS, the magnitude of ASM depends on the level of free TFs (middle panels). When the TF level is low, binding occurs on allele A (with high binding affinity) but stochastically in only a subset of DNA molecules. The overall proportion of low methylated reads (bound TFBS) reflects the steady state between dissociation and binding rates, defined by the concentration of the TF. At the other end of the concentration curve (right panel), strongly overexpressed TFs can bind both high and low binding affinity sites, leading to protection of both alleles against methylation and a loss of ASM. **B**, Inter-individual variability and allele-switching at ASM loci can be explained by a haplotype effects, in which multiple SNPs rather than a single SNP, or a dominant SNP in weak LD with the scored index SNP, dictate the ASM. This situation is “pseudo-switching”. **C,** Since most ASM SNPs found in this study can potentially disrupt multiple TF motifs, a TF competition model can explain bona fide allele-switching. This model appears to apply more often in cancer cells, which show a high frequency of ASM allele-switching in this study and are known to frequently over-express oncogenic TFs (e.g. c-MYC; **Fig. 5**).

Fig. S18

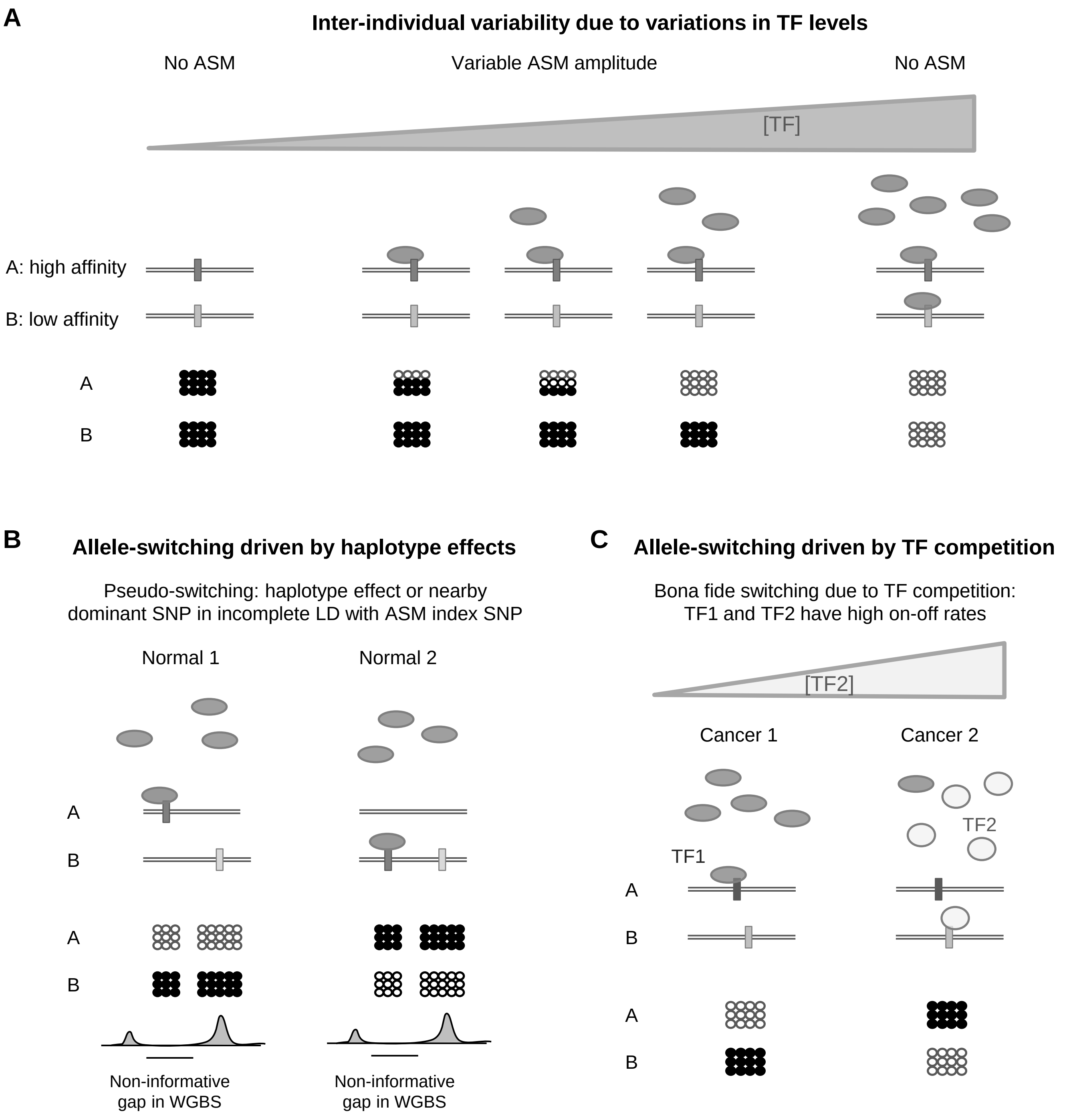
A
Inter-individual variability due to variations in TF levels
No ASM
Variable ASM amplitude
No ASM
[TF]
A: high affinity
B: low affinity
A
B
B
Allele-switching driven by haplotype effects
Pseudo-switching: haplotype effect or nearby dominant SNP in incomplete LD with ASM index SNP
Normal 1
Normal 2
A
B
A
B
Non-informative gap in WGBS
Non-informative gap in WGBS
C
Allele-switching driven by TF competition
Bona fide switching due to TF competition: TF1 and TF2 have high on-off rates
[TF2]
Cancer 1
Cancer 2
TF2
TF1
A
B
A
B

Fig. S19. The percentage of ASM loci that show switching behavior in cancers is smaller when considering only loci for which ASM is also detected in non-cancer samples

Graph showing the percentage of switching ASM loci in cancer and non-cancer samples as a function of the number of non-cancer samples where ASM is seen. For ASM loci in cancer, the x=0 data point corresponds to the percentage of switching among cancer-only ASM loci, while the subsequent data points show a decrease in switching among ASM loci found in both cancer and normal as the number of non-cancer samples (in addition to the cancer samples) showing ASM increases. As a comparison, the percentage of switching among ASM loci found in non-cancer samples is low and independent of the total number of samples showing ASM.

Fig. S19

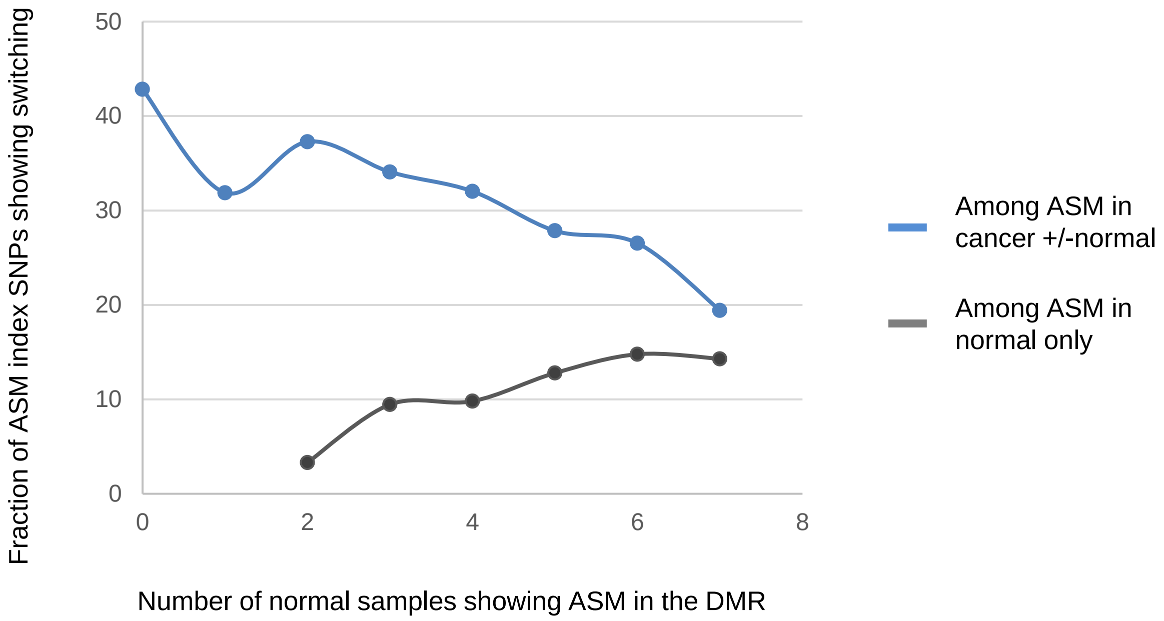
Fraction of ASM index SNPs showing switching
50
40
30
20
10
0
0
2
4
6
8
Number of normal samples showing ASM in the DMR
Among ASM in cancer +/-normal
Among ASM in normal only

Fig. S20. Examples of haplotype blocks defined by stringent and lenient parameters

Example of haplotype blocks on chromosome 5 using the Gabriel et al. approach based on confidence interval of D' values, with stringent (top) and lenient parameter (bottom). The lenient parameters, with relaxed D-prime confidence intervals and historical recombination rate (Methods), lead to haplotype blocks with larger sizes. Graphs were generated using Haploview with 1000 Genome data.

Fig. S20

Gabriel et al. (stringent) criteria

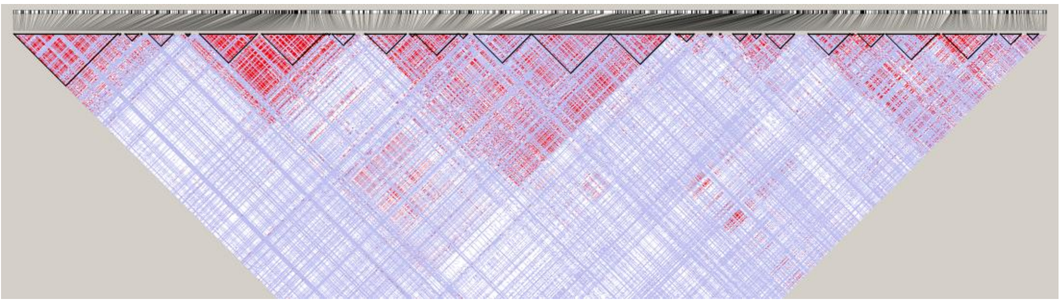

“Relaxed” criteria

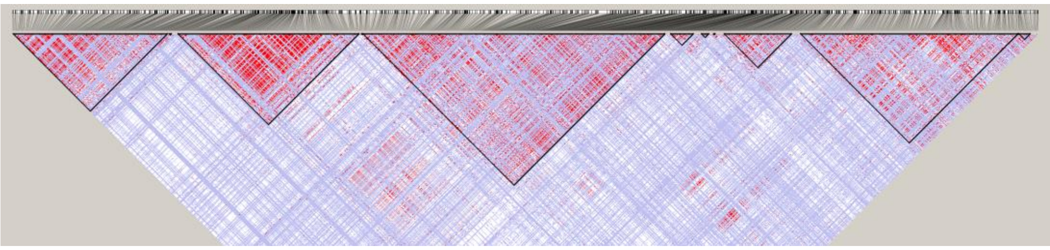

Fig. S21. Utility of D' and R-square parameters for assessing candidate disease-associated rSNPs

Example of D' (left) and R^2 (right) values between GWAS SNP rs710987 and all SNPs within 200 kb. The GWAS SNP is in red and ASM SNPs are in blue. The lenient haplotype block borders are shown in dashed green. The D' graph confirms that most of the SNPs within the block (including the ASM SNPs) exhibit high D' with the GWAS SNP and in this regard are in strong LD with it. The R^2 graph of the same window and SNPs shows that only a small subset of the SNPs in LD also exhibits high R^2 values, because even among SNPs in perfect LD only those with similar allele frequencies are expected to have high R^2 values. A complete understanding of disease associations, including possible effects of more than one rSNP in the same haplotype block, requires extending the identification of rSNPs to those in strong LD with the GWAS peak SNP based on D', even without high R^2 values.

Fig. S21

A

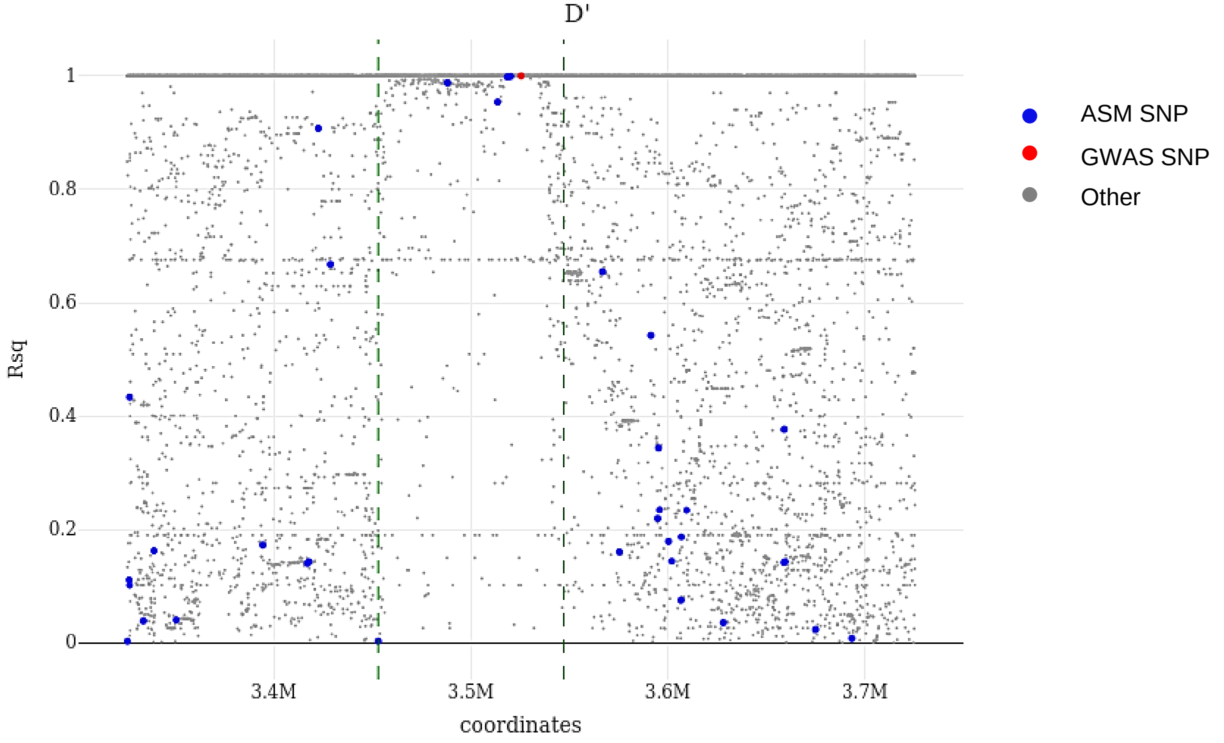

D'
1
0.8
0.6
0.4
0.2
0
Rsq
3.4M
3.5M
3.6M
3.7M
coordinates
ASM SNP
GWAS SNP
Other

B

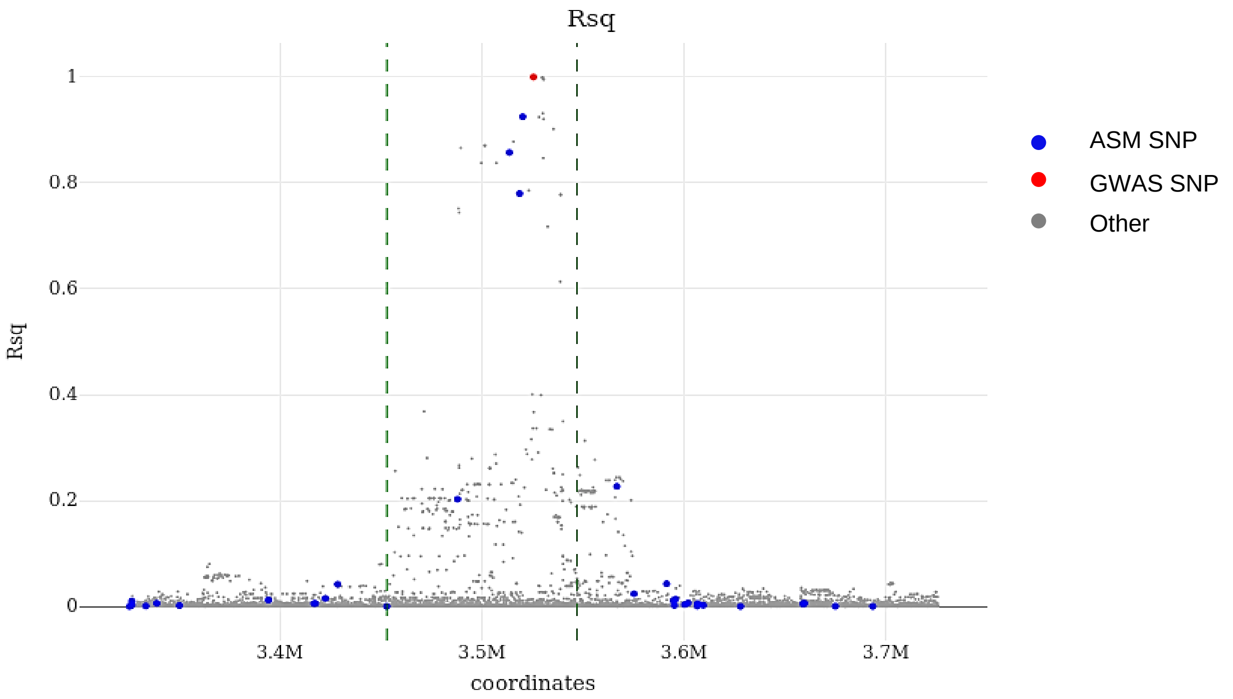

Rsq
1
0.8
0.6
0.4
0.2
0
Rsq
3.4M
3.5M
3.6M
3.7M
coordinates
ASM SNP
GWAS SNP
Other

Fig. S22. Additional examples of mechanistically informative disease associated ASM index SNPs: autoimmune and neuropsychiatric disorders

A, Map of the ASM region tagged by index SNP rs6603785 and located in an active enhancer region (color coded dark yellow) downstream of the *UBE2J2* gene on chromosome 1. ASM was observed in multiple blood cell types, including B cells. The ASM index SNP coincides with a GWAS peak SNP, associated with SLE ($p=9.0x10^{-6}$; O.R.=1.11) and hypothyroidism ($p=2.0x10^{-9}$; O.R. not listed). The SNP disrupts a MYC motif, with lower binding affinity and hypermethylation on the ALT allele, as predicted by the TF binding site occupancy model. **B**, Map of the ASM region tagged by index SNP rs2710323 and located in an active enhancer region (color coded in dark yellow) in the gene body of *ITIH1* on chromosome 3. ASM was observed in multiple blood cells, including T cells. The ASM index SNP coincides with a supra-threshold GWAS peak SNP for BMI measurements and multiple neuropsychiatric phenotypes including feeling nervous measurement, anxiety measurement, schizoaffective disorder, schizophrenia, and bipolar disorder (p-values and O.R. or Beta values in **Additional file 13: Table S12**). The SNP disrupts an ELF1 motif, with lower binding affinity and higher methylation, as predicted, on the REF allele. For this occurrence, the motif maps the negative strand and is reported from 3’ to 5’.

Fig. S22

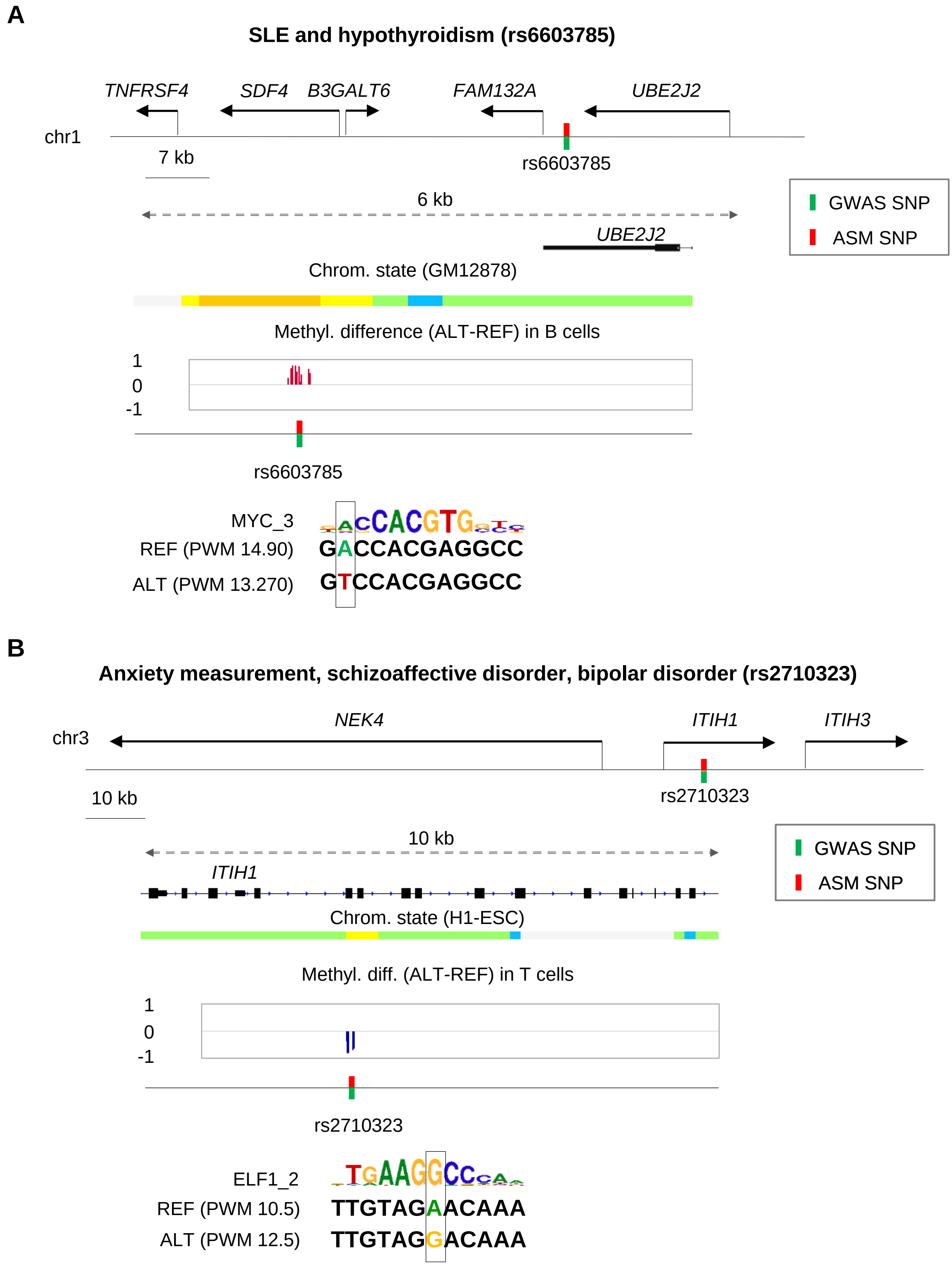
A
SLE and hypothyroidism (rs6603785)
TNFRSF4
SDF4
B3GALT6
FAM132A
UBE2J2
chr1
7 kb
rs6603785
6 kb
GWAS SNP
ASM SNP
UBE2J2
Chrom. state (GM12878)
Methyl. difference (ALT-REF) in B cells
1
0
-1
rs6603785
MYC_3
REF (PWM 14.90)
GACCACGAGGCC
ALT (PWM 13.270)
GTCCACGAGGCC
B
Anxiety measurement, schizoaffective disorder, bipolar disorder (rs2710323)
NEK4
ITIH1
ITIH3
chr3
10 kb
rs2710323
10 kb
GWAS SNP
ASM SNP
ITIH1
Chrom. state (H1-ESC)
Methyl. diff. (ALT-REF) in T cells
1
0
-1
rs2710323
ELF1_2
REF (PWM 10.5)
TTGTAGAACAAA
ALT (PWM 12.5)
TTGTAGGACAAA

Fig. S23. Additional examples of mechanistically informative disease associated ASM index SNPs: breast cancer and lymphoma

A, Map of the ASM region tagged by SNP rs3806624 and located in a poised promoter region (color coded purple) of the *EOMES* gene on chromosome 3. ASM was observed in DLBCL and in GBM, with allele switching between the two cancer types. The ASM index SNP coincides with a GWAS peak SNP for Hodgkin lymphoma ($p=1.0x10^{-12}$; O.R.=1.26) and is in strong LD with GWAS peak SNP rs9880772 associated with chronic lymphocytic leukemia ($p=3.0x10^{-11}$; O.R.=1.19), as well as with multiple myeloma (**Additional file 12: Table S11**). The SNP disrupts multiple motifs, including a BATF motif (lower binding affinity and higher methylation on the ALT allele) and a MAZ motif with opposite disruption of the binding affinity (lower binding affinity and higher methylation on the REF allele). A second ASM SNP nearby (rs2581199) is also located in poised chromatin, and it disrupts EGR2_1 and AP1_disc5 motifs. **B**, Map of the ASM region tagged by index SNP rs61837215 and located in the active promoter region (color coded red) of the *SEPT7P9* pseudogene (nearest coding gene, *ZNF37A*) on chromosome 10. ASM is observed in multiple myeloma cells and in normal B cells. The index SNP is in strong LD with GWAS peak SNP rs2754412 associated with breast cancer ($p=6.0x10^{-7}$; Beta=+.031). The ASM index SNP disrupts an ELF1_2 motif, with lower binding affinity and higher methylation on the REF allele, as predicted by the TF binding site occupancy model.

Fig. S23

A

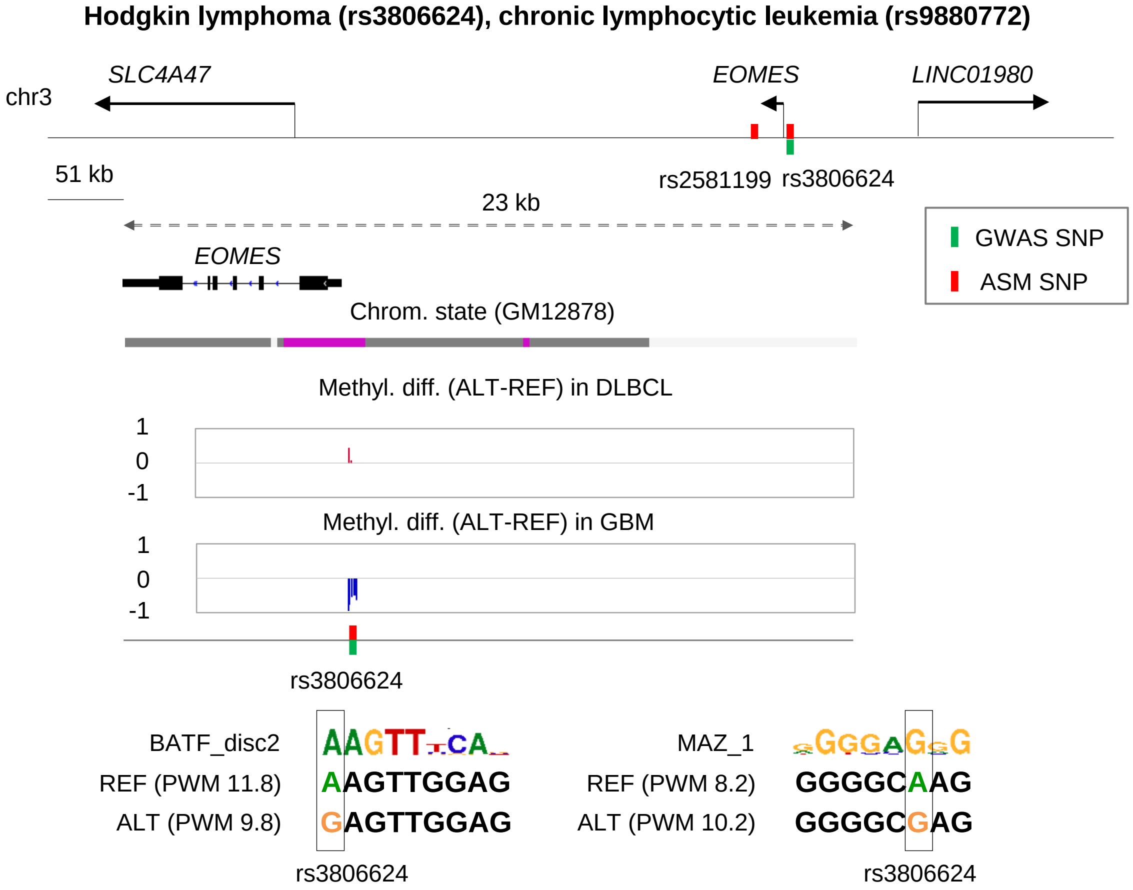
Hodgkin lymphoma (rs3806624), chronic lymphocytic leukemia (rs9880772)
SLC4A47
EOMES
LINC01980
chr3
51 kb
rs2581199
rs3806624
23 kb
GWAS SNP
ASM SNP
EOMES
Chrom. state (GM12878)
Methyl. diff. (ALT-REF) in DLBCL
1
0
-1
Methyl. diff. (ALT-REF) in GBM
1
0
-1
rs3806624
BATF_disc2
REF (PWM 11.8)
ALT (PWM 9.8)
AAGTTGGAG
GAGTTGGAG
rs3806624
MAZ_1
REF (PWM 8.2)
ALT (PWM 10.2)
GGGGCAAG
GGGGCGAG
rs3806624

B

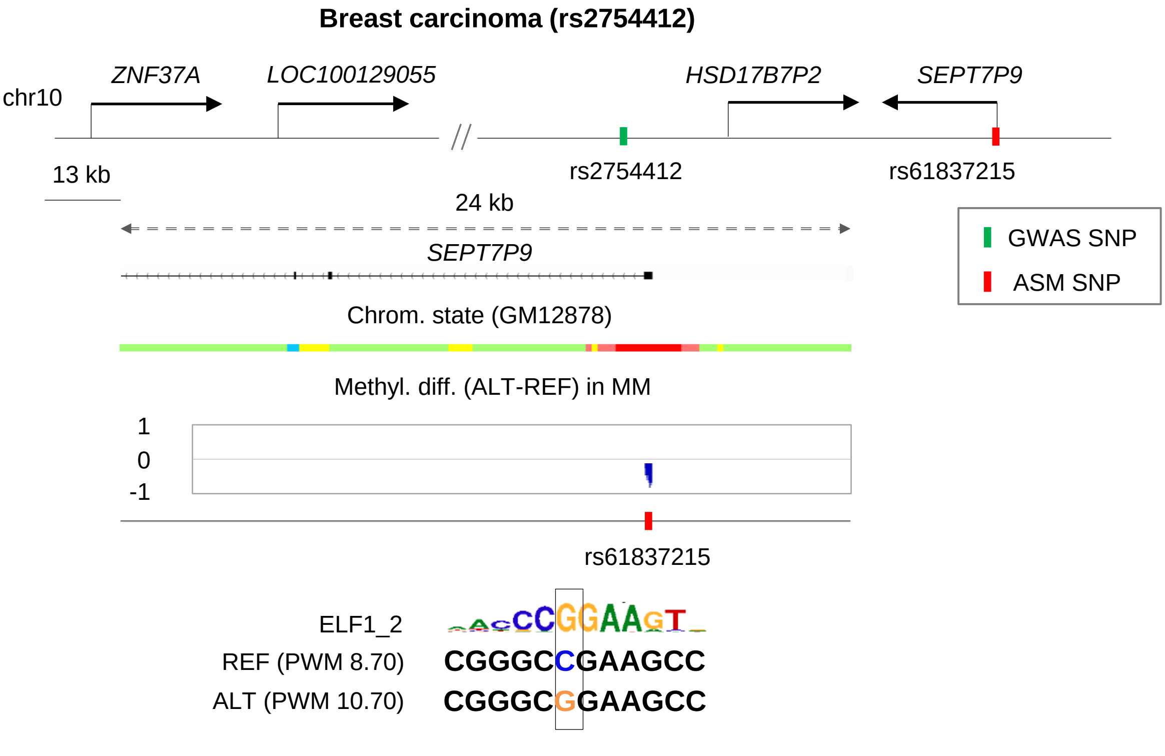
Breast carcinoma (rs2754412)
ZNF37A
LOC100129055
HSD17B7P2
SEPT7P9
chr10
13 kb
rs2754412
rs61837215
24 kb
GWAS SNP
ASM SNP
SEPT7P9
Chrom. state (GM12878)
Methyl. diff. (ALT-REF) in MM
1
0
-1
rs61837215
ELF1_2
REF (PWM 8.70)
ALT (PWM 10.70)
CGGGCCGAAGCC
CGGGCGGAAGCC

Fig. S24. ASM loci displayed as annotated genome browser tracks

Tracks of high confidence ASM loci are provided in UCSC browser format (see Availability of Data). ASM is color coded in blue scale for negative direction ASM (hypomethylation of ALT allele compared to REF allele on average across all ASM samples) and positive direction ASM is in red scale (hypermethylation of ALT allele compared to REF allele). Information about the index ASM SNP is displayed by clicking on the SNP (box). Reported information includes sample-aggregated information on the ASM-DMR and the index SNP, sample-specific information on ASM strength (p-value and methylation difference), the two classes of polymorphic motifs disrupted by the index SNP (i.e. enriched among ASM and/or with binding affinity-methylation correlation). Motif logo and sequences of the two alleles at the motif occurrences, generated using atSNP, are displayed by clicking on the motif name. Additional annotations of ASM index SNPs are in **Additional file 3: Table S2**.

Fig. S24

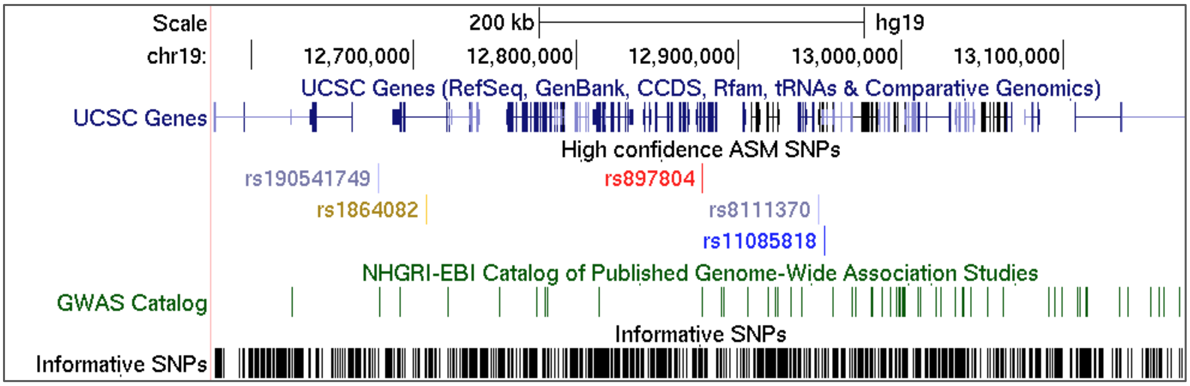

Custom Track: ASM SNPs

High confidence ASM SNPs

Item: rs897804
Score: 884
Position: chr19:12876964-12876964
Band: 19p13.2
Genomic Size: 1
View DNA for this feature (hg19/Human)

ID: chr19:12876798-12877138

General information about the ASM index SNP

ASM information for index SNP : rs897804

DMR infomation

- **DMR coordinates :** chr19:12876798-12877138
- **DMR overall rank :** 1
- **DMR strength rank :** 5
- **DMR confidence rank :** 45
- **SNP associated with DMR :** rs897804

Information about the ASM DMR

SNP level infomation

- **SNP overall rank :** 1
- **SNP strength rank :** 5
- **SNP confidence rank :** 45
- **Nb. samples with ASM :** 21
- **Nb. heterozygous samples :** 21
- **Switching ASM :** No

Ranking of the ASM index SNP

- **SNP color code :**

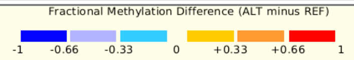

Color code for fractional methylation difference

Sample ID	Cancer status	Cell/Tissue	Methylation Difference	FDR	Nb. CpGs with ASM	Nb. covered CpGs	Sequencing platform
Sample 101	Cancer	Multiple Myeloma	.9	6.6e-04	15	17	WGBS
Sample 12	Non-Cancer	B Cells	1	5.6e-05	18	18	WGBS
Sample 17	Non-Cancer	Bladder Epith Cells	1	2.5e-04	17	17	WGBS
Sample 22	Non-Cancer	Brain Frontal Cortex	.7	1.2e-19	25	26	WGBS

Samples with ASM

Polymorphic motifs for rs897804

Enriched polymorphic motif

Motif name	PWM score ALT allele	PWM score REF allele	Difference in PWM score	FDR for the difference in PWM score
ABF1_MA0570_1	10.6	12.5	-1.9	6.8e-03
BCL_disc10	4.6	4.1	.5	<5e-324
CREB3L1_2	10.7	12.7	-2	2.3e-02

Polymorphic motifs with methylation-binding affinity correlation

Motif name	PWM score ALT allele	PWM score REF allele	Difference in PWM score	FDR for the difference in PWM score
CTCF_1	14.8	15.7	-.8	4.5e-03
CTCF_MA0139.1	14.8	15.6	-.8	4.8e-03

Go to ASM SNPs track controls

Data last updated: 2019-07-26

Polymorphic TF binding motifs enriched among ASM loci and disrupted by the ASM index SNP
