# Supplementary material for: Allele-specific DNA methylation is increased in cancers and its dense mapping in normal plus neoplastic cells increases the yield of disease-associated regulatory SNPs
Source: Genome Biol. 2020 Jun 29;21:153. doi: 10.1186/s13059-020-02059-3 (PMC7322865; doi:10.1186/s13059-020-02059-3)
Supplement: Supplementary file 14 — Additional file 14. Review history. [file 13059_2020_2059_MOESM14_ESM.docx]

Review History

**First round of review**

**Reviewer 1**

**Comments to author:**

Do et al present their work on allele-specific methylation combining large number of tissue types, conditions and experimental approaches in an attempt to create a resource for functional validations of disease SNPs and gain insight into methylation patterns within and across disease states (cancer vs non-cancer) in a way that "no previous study has been able to do". Although their work is comprehensive with a bigger sample size than the recent Onichic et al paper (science 2018), I argue against that statement as the work lacks statistical rigor and careful validations to support their findings making the subsequent results section difficult to judge (as described in detail below). The main challenge is the highly variable dataset combining so many technical variables that is impacting the accuracy of ASM and that likely only reducing the power. I have outlined my concerns in detail below:

*	Page 4: The work includes a variety of samples from not only different tissues and conditions but also lib prep protocol, seq method (targeted or wgbs), seq protocol (PE and SE) seq instrument (HiSeq X vs 2500) as well as sequencing metrics such as alignment rate (some samples are aligned at 63% whereas others at 93%). It is well known that these technical variables and batch effects significantly reduce the power and it is unclear how the authors correct for it or take it into account in analysis or validations.

*	Analytical pipeline: in general, the overview figure (S1) is not very clear. For instance, it indicates that the cutoff for an ASM CpG is 5X across both alleles whereas the methods section says 5X per allele, the "Allele B coverage > 20 and < 80 of total coverage" refers to % I assume but needs to be clarified. The section "Identification & ranking of ASM DMRs" needs clarification. It is also unclear if the genome was phased in order to use larger windows than the reads per se. the method specifies the use of 2kb window including multiple reads but also not specifying if phased genomes were used. A recent paper in Science (2018) by Onuchic et al with ~50 methylomes uses 30% cutoff as ASM whereas the present paper has 20% as the cutoff and it's unclear the reason for using this rather loose criteria especially with the very variable data sets (i.e. technical factors). It is also well known that the genome has regions that are problematic as stated by the ENCODE consortium due to anomalous, unstructured, or high signal in NGS data and often referred to as blacklisted regions. The authors should ensure that these regions are filtered out. Finally, the authors should make sure that highly covered reads from the end of the distribution are removed as these will significantly impact ASM calling.

*	Page 5: The authors don't have to spell out ALLL the different cell types used as everything is reported in Stable1.

*	Page 5: The authors report that five samples were tested across the two platforms (SureSelect and WGBS) but I count this only to four as one of the samples are CD3 sorted vs PBL so can't be counted as a technical replicate due to highly cell-specific nature of methylation patterns.

*	Page 6: As mentioned earlier figures are not very clear even when taken the legend text into account. In SFigure 2A, what are the % referring to in the venn diagram: 1 and 3% for SureSelect and 3 and 96% for WGBS. The authors are spelling out all the sample types instead of clarifying what the numbers are referring to. This figure needs substantial rework.

*	Page 6: The authors report a set of $11k recurrent ASM where 2 or more samples show the ASM without specifying or reporting whether these were mostly tissue-specific or tissue-shared. The information is indeed listed in Stable2 but only as concatenated sample names and not highlighted as a finding. As mentioned before, methylation is very tissue specific so I would make sense that a subset of these ASM are seen only in one tissue/cell type. Dividing them into tissue category would also facilitate the overlap with chromatin marks as discussed in more detail below. It is also notable that ~80% of the 11K ASM are seen in only 3 or 2 samples despite their total size of ~100 samples which the authors should comment on.

*	Page 7: the authors should specify the exact number of imprinted loci that were informative in SureSelect vs WGBS instead of the broad statement of "~100 imprinted loci " and report the validation rate in each data set. The text that follows indicates 35 loci in total that were validated but based on Table S4 it looks like it was 56 loci (?) but not whether it was discovered in SureSelect or WGBS or both remain unclear. This is important to ensure sample accuracy across platforms. The authors also report 15 extreme ASM loci (but with no further follow-up) but in case readers are interested in pursuing further studies the authors should consider adding the genome coverage of the CpGs in Table S5 to make sure they are not outliers due to highly covered regions.

*	Page 8: the authors present a "random" sample to visualize the "concordance" of results across platforms which is fine but more importantly they should perform systematic validations across the four samples where both methods were applied. The authors fail to do this which needs to be addressed, i.e. systematic comparisons of ASM detected in biological replicates and discovery vs replication rate. Related to this, it is unclear how the authors are indeed dealing with ASM detected in only 1 of the 2 replicates for the four samples with overlapping methods.

instead, the authors report the following based on one of the samples "Within the fraction of the genome that was adequately covered by both methods and contained informative SNPs, we found 1,590 (42.4%) shared ASM "hits". This is the global estimate across all samples, but the authors need to show the replication or correlation of ASM within a sample for the 4 samples assessed across platforms.

The authors go on and report a set of 18 ASM that are selected for further validation by BS-Seq again without justification what this random number of 18 is based on. Top 10% or top 1% would have been more valid or using a statistical cutoff etc. In addition, the use of "selected 18 ASM DMRs" is not randomly selected as only ~30% of the 18 replicated ASM are from the set of ASM seen in only 3 or 2 samples which is representing the vast majority (80%) of the 11K detected ASM. The authors have to provide further evidence that the replication rate of 83% is observed when more accurately selecting loci to validate. These types of flaws make the subsequent section more difficult to judge.

*	Page 12: Chromatin states (especially active enhancer states) are known to be tissue specific as shown by the NIH RoadMap consortium and others and more recently by the Onichic et al (Science 2018) who reports eight assays in most of the samples used for AI detection: WGBS, RNA-seq, and ChIP-seq for 6 different histone marks (H3K4me3, H3K4me1, H3K36me3, H3K27me3, H3K9me3, and H3K27ac). This tissue-targeted approach for ASM validations are used by other ASM work as well (Cheung et al 2017 Genome Biology). Here, the authors fail to present the source of the chromatin layer and seem not to take tissue-specificity into account as highlighted above as well.

*	Page 13 and onwards: The canonical signal observed by authors including hypermethylation observed for motif disruption is reassuring, but the earlier study by Onuchic et al. Science 2018 investigating ASM reported motif disruption with hypomethylation for substantial fraction of sites - this controversy begs for more detailed analyses - the datasets should be publicly available and overlap between these studies in terms of CpGs reported to harbor ASM is high. This is also a general comment that the authors should use this dataset for more proper validations (in greater detail than a short note in the discussion section).

*	Page 19: The authors seek to find ASM SNPs in LD with GWAS hits but it should be noted that they have no power to detect true colocalization of ASM causal SNP and disease SNP. With the lenient criteria (even using D' rather than r2) for detecting overlap it is likely there is no evidence of enrichment of GWAS SNPs among ASM variation and the whole section remains descriptive.

**Reviewer 2**

**Comments to author:**

The study includes multiple valuable contributions centered on the theme of allele-specific methylation (ASM): a high-value ASM dataset; novel mechanistic insights into ASM; the first comprehensive comparison of mechanisms of ASM in normal and cancer cells; a demonstration of the utility of ASM mapping as an adjunct to GWAS studies to help narrow down the list of SNPs to the regulatory variants that may cause disease.

The dataset includes whole genome bisulfite sequencing (WGBS, all ~30M CpGs in the human genome) 61 non-cancer tissues of various types from diverse individuals and 14 cancer methylomes representing three cancer types. This greatly expands previous genome-wide methyl-seq targeted profiling using Agilent SureSelect targeted capture technology (~3M CpGs covered) previously reported by Tycko laboratory. This new study identifies >11,000 high-confidence ASM showing > 20% allelic methylation difference in at least two distinct methylomes and performs validation to establish specificity.

The study provides mechanistic insights into ASM, particularly the chromatin states that are enriched and depleted for ASM in normal and cancer cells.

The ASM map and analysis of allele-specific methylation provide the first comprehensive comparison of mechanisms of ASM in normal and cancer cells. Specifically, the study demonstrates several-fold increase in ASM in cancer cells due to genome-wide hypomethylation and focal methylation in polycomb-regulated loci, consistent with epigenetic regulation in cis.

One particularly intriguing finding is the excess of allele switching observed in cancer. This finding provides a new perspective on epigenetic instability of cancer cells.

The study demonstrates utility of ASM mapping as an adjunct to GWAS studies by identifying >800 candidate causal SNPs within loci implicated by GWAS in a spectrum of human common diseases including cancer, neuropsychiatric and immune diseases.

The paper lacks some information that is important for interpreting the reported findings and also raises some questions that, if answered, would significantly strengthen reader's confidence in the results:

1. Regarding the excess of allele switching observed in cancer, TF deregulation and global de-methylation in LCLs and cancer cells randomly targeting one of the two chromosomes are offered as explanations. Have possible effects in cis of cancer mutations, including structural rearrangements been considered?

2. It is not clear of the Agilent dataset was used for ASM discovery and of so, what was the effect of the target selection inherent in the Agilent dataset?

3. Presumably due to the absence of genome sequences of donors, only a fraction of heterozygous sites was examined (CpG sites being excluded). How many? Would the selection of only that subset of hets introduce ascertainment bias? How was the bias controlled?

4. Apparently, because genomes have not been sequenced, only the "index hets" involving minor alleles with relatively high frequency have been assessed. How did this bias away from variants that have large effects and therefore tend to have low frequency due to purifying selection affected analyses?

5. The "index hets" are identified by intersecting WGBS data with a list of SNPs from the public domain. How was the list of SNPs compiled?

6. How was the bias toward better mapping of reads with major alleles (reference sequence bias) been addressed for allele-specific ChIP-seq (p.15-16).

7. Could the absence of "reverse bias" where the disruption of TF binding decreases methylation be explained by any ascertainment differences between the present and previous study? If so, what are the potentially most relevant ones?

8. Is there evidence that the ASM for GWAS regulatory SNPs associates with allelic transcription in cis in the etiologically relevant cell types?

Other relatively minor issues:

p.5, Line 52: Which glia and neuron samples are being referred to?

The type of GBM (if CIMP subtype, it should be stated as it is relevant for interpreting DNA methylation results).

p.5 First paragraph: Unclear phrase: "non-CpG containing polymorphic CTCF motif instances".

p.8, Line 20-25: Unclear statement.

The term "Human Epigenome Project" was used as an incorrect reference to the "NIH Roadmap Epigenomics Project"

p.14. Lines 43-48: How many motifs are found in the randomized set, compared to the 860 found in real data? This number will help interpret the result if spelled out in the main text.

Supplemental Excel tab "S2 ASM All excludes imprinted" shows more than 13,200 ASM SNPs whereas the paper reports less than 13,000; why the difference?

In the supplemental spreadsheet, "rsIDs" refer to polymorphisms, not to individual alleles. Some important allele-specific information (e.g., methylation levels in specific wgbs datasets) may be communicated by reference to specific alleles (e,g., using the ClinGen Allele Registry to obtain the IDs of specific alleles).

**Authors Response**

**Point-by-point responses to the reviewers’ comments:**

*We appreciate the very useful comments from both Reviewers, and we address their questions and suggestions point-by-point as follows:*

Reviewer #1: ... The main challenge is the highly variable dataset combining so many technical variables that is impacting the accuracy of ASM and that likely only reducing the power. I have outlined my concerns in detail below:

*	Page 4: The work includes a variety of samples from not only different tissues and conditions but also lib prep protocol, seq method (targeted or wgbs), seq protocol (PE and SE) seq instrument (HiSeq X vs 2500) as well as sequencing metrics such as alignment rate (some samples are aligned at 63% whereas others at 93%). It is well known that these technical variables and batch effects significantly reduce the power and it is unclear how the authors correct for it or take it into account in analysis or validations.

*We understand and appreciate this reviewer’s concern about the three different sequencing facilities and library prep kits for the WGBS. To address this question about possible “facility dependent” results, we have added 8 new samples, all with WGBS performed at the Roswell Park Cancer Institute (RPCI) genomics facility, which was the most intensively utilized facility in our first submission of the manuscript (MS).*

*In the resulting enlarged series of samples (25 Agilent; 81 WGBS) we now have 56 WGBS specifically from the RPCI facility. Using this expanded “RPCI facility only” dataset, we have confirmed all the main findings from the first submission of our manuscript.*

*Here we show two such confirmations – for the gains of ASM in cancer vs normal and the dependence of this phenomenon on global genomic hypomethylation (Response Fig. I), and for the consistently negative slopes of the correlations of TF binding site affinity scores with CpG methylation within ASM loci, exemplified by ETS2_1 here (Response Fig. II), but pertaining to the complete set of correlated motifs, as detailed in Table S8 of our MS.*

*The differences between our conclusions and those of Onuchic et al., for a subset of TFBS classes, including ETS2_1 that we illustrate here, are in fact explained by the greater stringency of our ASM calling, in which we require multiple ASM CpGs, and apply a false discovery rate<0.05, in order to call an ASM DMR (see our detailed explanation below, and the flow chart and diagram in Fig. S1A, B).*

*Our PCA result (Fig. S3A) and the substantial overlap of the WGBS and Agilent SureSelect results for loci sequenced to adequate depth on both platforms (Fig. S3B, C) further support the validity of the combined dataset. In addition, as illustrated in the newly added Figure S5A, B, samples analyzed by WGBS using two different library preps, at two different facilities, showed good correlations of net methylation and methylation differences between alleles. Moreover,* *as noted above, by adding 8 new WGBS samples (two cancer and 6 non-cancer) and then using the expanded data from a single facility (RPCI-only; 56 samples) we have confirmed all the main findings of our manuscript.*

*The high rate of validations of ASM by targeted bis-seq (now expanded to more loci spanning the mid- and lower-range of ASM ranking scores, as requested) further support the high true-positive rate of our ASM calling. We have been careful in the original version of the MS and in this revision to emphasize that the resulting maps of ASM are dense and useful, but still non-saturating. That is, there are definitely “false negatives” in our dataset (as in all other published ASM mapping studies). Such gaps will be filled in over time by adding even more samples (more heterozygous calls), and greater sequencing depth.*

*Regarding alignment rates, we agree with the reviewer’s suggestion, and we have excluded the three samples (annotated in Table S1) with alignment rates <75%, so all the samples included in our analyses now have alignment rates >75% (median 83%), which is in line with the rates in other published WGBS studies.*

*Regarding the issues of potential batch effects and clinico-demographic biases, our ASM calling is a within-sample approach, where for each assessed heterozygous SNP observed in each sample, one allele (the REF allele) is the control for the other (ALT) allele. In that sense our ASM calling is fundamentally controlled for potential batch effects and clinico-demographic biases in a similar way as any other type of within-sample paired analysis. Compared to previous studies of ASM, the advantage of our dataset is the inclusion of many different individuals, which increased the set of tested informative SNPs, included rarer SNPs, allowing an assessment of inter-individual variability.*

*	Analytical pipeline: in general, the overview figure (S1) is not very clear. For instance, it indicates that the cutoff for an ASM CpG is 5X across both alleles whereas the methods section says 5X per allele, the "Allele B coverage > 20 and < 80 of total coverage" refers to % I assume but needs to be clarified. The section "Identification & ranking of ASM DMRs" needs clarification.

*We appreciate these comments and we have clarified our flow chart in Fig. S1A and added a diagram, in the new Fig. S1B, to more clearly show our ASM criteria.*

*It is also unclear if the genome was phased in order to use larger windows than the reads per se. the method specifies the use of 2kb window including multiple reads but also not specifying if phased genomes were used. A recent paper in Science (2018) by Onuchic et al with ~50 methylomes uses 30% cutoff as ASM whereas the present paper has 20% as the cutoff and it's unclear the reason for using this rather loose criteria especially with the very variable data sets (i.e. technical factors).*

*We agree that for ASM calling the use of PE reads is of most importance, since the queried index SNP and its nearby CpGs need to be phased. This situation is improved by longer reads and by PE reads since both reads from a pair can be phased together, allowing larger insert size for phasing the SNPs and CpGs. Therefore, all the samples in our multi-year study have been sequenced using the longest size Illumina PE mode that was readily available at each time that the data were generated. Regarding the 20% methylation difference cutoff for calling ASM, we wish to emphasize that we require it at multiple clustered CpGs within the sequence contig (see Fig. S1A, B), as well as a false discovery rate<0.05. So, our criteria for ASM are in fact more stringent in terms of p-value as well as effect size threshold than the criteria used by Onuchic et al., who used a 30% methylation difference cutoff but called ASM even when only a single CpG showed this asymmetry, and used a more lenient FDR<0.10.*

*It is also well known that the genome has regions that are problematic as stated by the ENCODE consortium due to anomalous, unstructured, or high signal in NGS data and often referred to as blacklisted regions. The authors should ensure that these regions are filtered out. Finally, the authors should make sure that highly covered reads from the end of the distribution are removed as these will significantly impact ASM calling.*

*We appreciate and agree with the reviewer’s comment and we have now excluded all SNPs overlapping blacklisted regions. We would like to highlight that being aware of potential SNP calling issues in these problematic regions, we used UCSC annotations from DbSNP (Methods section) to filter out heterozygous SNPs with multiple mapping. Moreover, and importantly, since our dataset includes many different individuals, we were able to test Hardy-Weinberg equilibrium for each ASM SNP and excluded any SNPs that were out of equilibrium. These two additional filtering steps had in fact excluded most of the problematic SNPs in our initial submission of the MS. Lastly, as described and now clarified in the Methods section, to exclude ambiguous repetitive regions we had already set the maximum coverage for SNP calling at 200X, and the coverage of all our high confidence ASM index SNPs met this criterion.*

*	Page 5: The authors don't have to spell out ALL the different cell types used as everything is reported in Stable1.

*We agree and we have moved these details to the Methods section.*

*	Page 5: The authors report that five samples were tested across the two platforms (SureSelect and WGBS) but I count this only to four as one of the samples are CD3 sorted vs PBL so can't be counted as a technical replicate due to highly cell-specific nature of methylation patterns.

*This comment is not true; as shown in Table S1, 5 samples were analyzed across SureSelect and WGBS:*

*-1 brain from subject 44: sample 21 (SureSelect) and sample 22 (WGBS)*

*-1 T cell prep from subject 23: sample 67 (SureSelect) and sample 68 (WGBS)*

*-1 T cell prep from subject 25: sample 70 (SureSelect) and sample 71 (WGBS)*

*-1 T cell prep from subject 27: sample 74 (SureSelect) and sample 75 (WGBS)*

*-1 lymphoblastoid line (GM12878) from subject 33: sample 35 (SureSelect), sample 36 (WGBS).*

*	Page 6: As mentioned earlier figures are not very clear even when taken the legend text into account. In SFigure 2A, what are the % referring to in the venn diagram: 1 and 3% for SureSelect and 3 and 96% for WGBS. The authors are spelling out all the sample types instead of clarifying what the numbers are referring to. This figure needs substantial rework.

*We understand this problem and we have updated Figure S2A and the legend to clarify these percentages. The percentages represent:*

*1%= A / (A ∪ B)*

*3%= (A ꓵ B) / (A ∪ B)*

*96%= B / (A ∪ B)*

*With A ∪ B =A + A ꓵ B + B=100%*

*In other words, 1% of the total number of informative SNPs in our dataset were found only in SureSelect samples, 3% are found in both WGBS and SureSelect and 96% only in WGBS samples, which means that 4% (3 + 1) of the SNPs were informative in SureSelect and 99% (96 + 3) in WGBS.*

*	Page 6: The authors report a set of 11k recurrent ASM where 2 or more samples show the ASM without specifying or reporting whether these were mostly tissue-specific or tissue-shared. The information is indeed listed in Stable2 but only as concatenated sample names and not highlighted as a finding. As mentioned before, methylation is very tissue specific so I would make sense that a subset of these ASM are seen only in one tissue/cell type. Dividing them into tissue category would also facilitate the overlap with chromatin marks as discussed in more detail below.

*Since Table S2 is designed to provide annotations of the index ASM SNPs (e.g. TFBS disruption, GWAS LD, ASB etc.), we created this table with each line representing an ASM index SNP. Thus, the unique identifiers in Table S2 are the ASM index SNPs, sometimes with multiple index SNPs in a single ASM DMR, as expected.*

*To not multiply the number of columns excessively, many columns contain concatenated information, including tissues and samples where the index SNP was heterozygous (i.e. informative) and associated with ASM. This format facilitates quick checks, such as if a given ASM SNP was observed only in one tissue while multiple tissues were informative, which would suggest of tissue-specificity, or if ASM was found only in one tissue but only one tissue was informative, which is inconclusive regarding tissue-specificity. In addition, to help in querying tissue specificity, for each SNP, the number of samples and tissues where the SNPs were informative and ASM was found are reported. Furthermore, we provided a detailed bed file that can be uploaded to the UCSC browser (see Fig. S24) to display, for each ASM SNP, in long format, the ASM parameters for each sample and tissue, as well as the by-sample bedgraph files, corresponding to our GEO submission.*

*Regarding our ability to address tissue specificity, only SNPs informative in multiple tissues can be assessed. In addition, since ASM shows inter-individual variability, to correctly analyze ASM tissue specificity, each tissue needs to be informative in multiple heterozygous individuals. Therefore, to answer the reviewer’s comment, we assessed ASM tissue specificity using the informative subset of 5,394 ASM SNPs where at least 3 samples per tissue and at least 2 different tissues were informative. We found 1,519 ASM SNPs (i.e. 28 % of the loci that could be tested) where the results suggest tissue specificity or at least some degree of tissue restriction.*

*However, these results from the overall series need to be taken with caution since they are highly dependent on the number of informative biological samples for each ASM DMR. We therefore also performed pairwise correlations between ASM using our set of paired samples from the same individuals, which suggest that some tissue specific ASM DMRs are actually subthreshold in the non-ASM tissue (we are showing below one of the correlations between cell types from the same subject, the other correlation showing similar results). These analyses of tissue specificity, while still far from saturating, are now briefly highlighted in the manuscript.*

It is also notable that ~80% of the 11K ASM are seen in only 3 or 2 samples despite their total size of ~100 samples which the authors should comment on.

*We agree this question is important. ASM inter-individual variability needs to be assessed taking account the number of heterozygous/informative samples to distinguish ASM found only in 2-3 informative samples/out of only 2-informative samples vs. ASM found in 2-3 samples out of multiple informative samples from the same tissue. The first situation represents 2% of our ASM DMRs, which are simply enriched in rare index SNPs (i.e. not true inter-individual variability). For ASM loci where the SNP was informative in enough samples (at least 2 samples from the same tissue), we found between 9% to 17% of ASM SNPs for which ASM was detected in less than 50% of the samples (i.e. bona fide inter-individual variability).*

*Our sample set includes a large number of different individuals ( 75 vs 13 in Onuchic et al.), so our ASM calls by sample (rather than pooled sequencing data between multiple samples) was able to unravel this ASM individuality, which might turn out to be biologically and medically relevant for explaining inter-individual variability in the effects of disease - associated SNPs, potentially contributing to low Odds Ratios reported by GWAS. We have added this to the revised Discussion section.*

*	Page 7: the authors should specify the exact number of imprinted loci that were informative in SureSelect vs WGBS instead of the broad statement of "~100 imprinted loci " and report the validation rate in each data set. The text that follows indicates 35 loci in total that were validated but based on Table S4 it looks like it was 56 loci (?) but not whether it was discovered in SureSelect or WGBS or both remain unclear. This is important to ensure sample accuracy across platforms. The authors also report 15 extreme ASM loci (but with no further follow-up) but in case readers are interested in pursuing further studies the authors should consider adding the genome coverage of the CpGs in Table S5 to make sure they are not outliers due to highly covered regions.

*As discussed above, ASM calls between SureSelect and WGBS were well correlated. However, we have added additional columns that provide the sample count separately for Sure Select and WGBS.*

*To assess imprinting (ASM switching with a theoretical switching rate of 50%), enough samples showing ASM are required. Although we listed in Table S4 any ASM loci within 75 kb of a known imprinting genes, we only considered in the text the loci where ASM was found in more than 10 samples. We have added the average coverage per allele, as well as a count of samples stratified by platform in Table S5.*

*	Page 8: the authors present a "random" sample to visualize the "concordance" of results across platforms which is fine but more importantly they should perform systematic validations across the four samples where both methods were applied. The authors fail to do this which needs to be addressed, i.e. systematic comparisons of ASM detected in biological replicates and discovery vs replication rate. Related to this, it is unclear how the authors are indeed dealing with ASM detected in only 1 of the 2 replicates for the four samples with overlapping methods instead, the authors report the following based on one of the samples "Within the fraction of the genome that was adequately covered by both methods and contained informative SNPs, we found 1,590 (42.4%) shared ASM "hits". This is the global estimate across all samples, but the authors need to show the replication or correlation of ASM within a sample for the 4 samples assessed across platforms.

*As suggested, we have added a cross-facility comparison for our technical replicates on the same biological samples. Figure S5A shows good correlations in the net methylation readouts for these samples analyzed by the pairs of facilities, and Figure S5B shows that most of the ASM found in only one facility shows the same methylation difference trend (i.e. “direction” of the allelic methylation bias) in both facilities, but is sub-threshold in the data from one facility, in terms of p-value and/or number of ASM CpGs.*

*For WGBS samples assessed using different library preps as described in the Methods, we bioinformatically pooled the reads which give similar results as an average of the methylation estimates weighted for coverage.*

*For samples assessed both by SureSelect and WGBS, the technical replicates were kept separate. As shown in our cross-facility analysis (above), most of the “discordant” ASM hits were in fact simply subthreshold (in the same direction) in the other replicate. We therefore considered that ASM was present if observed in at least one of the technical replicates.*

*As noted above, in addition to these good correlations between the data from multiple facilities and multiple library construction methods, we have confirmed all our main findings from the first submission of the MS using data exclusively from the RPCI facility.*

The authors go on and report a set of 18 ASM that are selected for further validation by BS-Seq again without justification what this random number of 18 is based on. Top 10% or top 1% would have been more valid or using a statistical cutoff etc. In addition, the use of "selected 18 ASM DMRs" is not randomly selected as only ~30% of the 18 replicated ASM are from the set of ASM seen in only 3 or 2 samples which is representing the vast majority (80%) of the 11K detected ASM. The authors must provide further evidence that the replication rate of 83% is observed when more accurately selecting loci to validate. These types of flaws make the subsequent section more difficult to judge.

*We understand this concern, and we have added targeted bis-seq on loci from the mid- to low-ranked ASM range (with no selection bias). Our overall independent tests by targeted bis-seq now constitute a total of 27 loci, which will be one of the largest (maybe the largest) published sets of independent ASM validations. The results, namely 81% validation of ASM loci using biological samples outside the genome-wide primary series and 96% validation when we include targeted bis-seq validations on index samples from the genome-wide primary series, are described in the revised MS. These are strong and realistic “outside the series” and “inside the series” validation rates, particularly considering the substantial inter-individual variability in presence or absence of ASM.*

*	Page 12: Chromatin states (especially active enhancer states) are known to be tissue specific as shown by the NIH RoadMap consortium and others and more recently by the Onichic et al (Science 2018) who reports eight assays in most of the samples used for AI detection: WGBS, RNA-seq, and ChIP-seq for 6 different histone marks (H3K4me3, H3K4me1, H3K36me3, H3K27me3, H3K9me3, and H3K27ac). This tissue-targeted approach for ASM validations are used by other ASM work as well (Cheung et al 2017 Genome Biology). Here, the authors fail to present the source of the chromatin layer and seem not to take tissue-specificity into account as highlighted above as well.

*To answer this comment, we performed a cell type-specific enrichment analysis of chromatin states using Roadmap Epigenomics data and found similar results as from our non-tissue specific analysis. Further, we did not observe enrichment differences between tissues (added in Methods and Results sections). In this regard, our results are consistent with the Cheung et al 2017 findings and support the hypothesis that while ASM maps are tissue-specific, the underlying mechanisms appear to be largely shared across tissue and cell types.*

*	Page 13 and onwards: The canonical signal observed by authors including hypermethylation observed for motif disruption is reassuring, but the earlier study by Onuchic et al. Science 2018 investigating ASM reported motif disruption with hypomethylation for substantial fraction of sites - this controversy begs for more detailed analyses - the datasets should be publicly available and overlap between these studies in terms of CpGs reported to harbor ASM is high. This is also a general comment that the authors should use this dataset for more proper validations (in greater detail than a short note in the discussion section).

*The key point is that in our MS we only report strong correlations (FDR<0.05) and Rsq >0.4, while Onuchic et al. reported ASM vs. TF motif PWM correlations without p-value and goodness of fit criteria. As specified in their methods, they performed a preselection of TF classes from the set of TFs showing a correlation between PWM and constraint coefficient, which is an estimate of methylation entropy but not ASM. In fact, in addition to the 2 TF motifs with significant positive correlations reported in Table S8 positive we observed non-significant positive correlations for 482 TF motifs, including 82 TF motifs (corresponding to 57 TFs) with weak subthreshold (nominal p-value<0.05 and no Rsq cutoff) that we did not report since these instances do not pass our statistical criteria and we believe are less robust and would require further validations. Among the 45 TFs that were reported by Onuchic et al. as TF motifs with positive correlations to ASM, 30% showed a similar weak trend, but not statistically significant, in our dataset.*

*Most of the discordances between our findings and the Onuchic et al. findings concern ETS-family TF correlations (uniformly inverse correlations in our analysis, but some positive correlations in theirs). To investigate these differences, we performed allele-specific PWM scoring using our pipeline and ENCODE-JASPAR PWM, specifically on the set of ASM SNPs reported by Onuchic et al. By this approach we found an ELK3_2 TF affinity correlation graph with a positive slope, as published by Onuchic et al. However, this correlation was not significant (FDR=0.5) and showed a poor fit (Rsq=0.004). When we use our set of stringently defined ASM SNPs (see Fig. S1A, B for our criteria) our ETS-family TF motif correlations have negative slopes and are significant (For ELK3_2, Rsq=0.47, FDR=10-7).*

*In addition, as noted above, our ASB analysis for the set of our ASM SNPs that are found in the GM12878 cell line showed that the binding of ETS-family TFs in this cell line takes place on the low methylated allele in a large majority of occurrences genome-wide, supporting the negative correlation between ETS binding affinity and ASM.*

*Lastly and importantly on this topic, we would like to point out (and we state in the MS) that a good number of TF and CTCF motif classes have in fact given concordant results in our study and that of Onuchic et al. For some of these motifs (e.g. CTCF sites) our data show that the ASM signals are particularly strong, which can explain the concordance in findings, since the slopes of the X-Y plots are dominated by strong (and therefore statistically significant) ASM occurrences.*

*Our data have been submitted to NCBI GEO (GSE137287, GSE137879 linked by superseries GSE137880)*

*	Page 19: The authors seek to find ASM SNPs in LD with GWAS hits but it should be noted that they have no power to detect true colocalization of ASM causal SNP and disease SNP. With the lenient criteria (even using D' rather than r2) for detecting overlap it is likely there is no evidence of enrichment of GWAS SNPs among ASM variation and the whole section remains descriptive.

*In the first submission of our MS we had annotated each ASM index SNP for its relationship to GWAS peaks using both stringent and lenient criteria based on haplotype block algorithms (relying mostly on D-prime), as well as Rsq criteria. We have maintained that comprehensive approach in the revised MS (see Tables S2 and S3). Regarding GWAS enrichment, we observed a moderate but nonetheless significant and increasing enrichment in GWAS SNPs present in the same haplotype block using both lenient and stringent haplotype criteria (O.R=1.1; p-value=8x10-14 and O.R=1.2; p-value=2x10-15, respectively ), as well as in GWAS SNPs in LD with ASM SNPs with Rsq >0.8 ((O.R=1.4 ; p-value=10-47) and in ASM SNPs that precisely coincide with GWAS SNPs (O.R.=2, p-value p-value=10-42). This increasing enrichment associated with the increase of stringency strongly supports a genuine enrichment in GWAS SNPs among ASM index SNPs.*

Reviewer #2: The study includes multiple valuable contributions centered on the theme of allele-specific methylation (ASM): a high-value ASM dataset; novel mechanistic insights into ASM; the first comprehensive comparison of mechanisms of ASM in normal and cancer cells; a demonstration of the utility of ASM mapping as an adjunct to GWAS studies to help narrow down the list of SNPs to the regulatory variants that may cause disease.

*Several other positive remarks follow - below we turn to this reviewer’s specific questions.*

Q1:	Regarding the excess of allele switching observed in cancer, TF deregulation and global de-methylation in LCLs and cancer cells randomly targeting one of the two chromosomes are offered as explanations. Have possible effects in cis of cancer mutations, including structural rearrangements been considered?

*We appreciate this potentially important question about possible “de novo” gains of ASM due to somatic mutations in cancers. It is something we were working on for another paper, but since the data already look promising, we are including it here in the revised MS.*

*To examine this possibility, we utilized four multiple myeloma / normal peripheral blood B cell pairs from patients. Overall in this series, we found 338 somatic mutations associated with ASM out of 3,444 total somatic mutations, with a percentage of ASM among the somatic mutations ranging from 6 to 17%. While this percentage of de novo ASM among the somatic mutations is slightly higher than expected based on what we observed for ASM associated with germline SNPs (paired t-test p=0.03), the number of somatic mutation-associated ASM loci is obviously low compared to total ASM loci (i.e. the phenomenon seems to be non-random but it does not contribute very much to the overall gains of ASM in the cancers).*

*More informatively, among the 338 mutation-associated de novo ASM occurrences, 276 disrupted at least one TF binding motif, and we found 58 enriched TF polymorphic motifs. Of these, 90% also showed enrichment in the larger set of (germline) ASM index SNPs (OR=2.7, p-value=5x10-9). This result is meaningful in that it suggests a shared underlying mechanism between germline SNP-associated ASM and somatic mutation-associated de novo ASM.*

*In our series we do not see effects of copy number duplications on ASM frequency, the percentage of ASM by chromosome remaining similar for duplicated chromosomes compared to euploid chromosomes. However, we would not be surprised if some gains of ASM were to be found in aneuploid chromosomal regions of human cancers in future studies.*

Q2:	It is not clear of the Agilent dataset was used for ASM discovery and of so, what was the effect of the target selection inherent in the Agilent dataset?

*The Agilent SureSelect dataset was used (and it was useful) for ASM discovery; 3% of our total ASM index SNPs were detected in Agilent data, and many but not all of these ASM loci were also found by WGBS (on a largely different sample set). This low percentage is explained by the smaller set of informative SNPs in Agilent due to target selection, and the lower number of samples (as shown in Fig. S2A). In addition, the Agilent pulldown enriches for CpGs in*

*regulatory elements (promoters and enhancers), and we observed a higher percentage of ASM in Agilent compared to WGBS, which is consistent with the enrichment of ASM in regulatory elements that we described here and previously.*

Q3:	Presumably due to the absence of genome sequences of donors, only a fraction of heterozygous sites was examined (CpG sites being excluded). How many? Would the selection of only that subset of hets introduce ascertainment bias? How was the bias controlled?

*We would like to clarify that although we did not perform WGS, using bisSNP we performed genotyping directly on WGBS reads, using the entire set of rare and common SNPs reported in dbSNP147. We updated Figure S1 to clarify this point. In addition, using GM12878 genotyping data from the 1000 genome project, we found that >99% of the ASM SNPs detected in GM128778 from our WGBS were also genotyped by that project as heterozygous using WGS.*

*We excluded tri-allelic SNPs, SNPs with multiple mapping, SNPs with coverage >200x (i.e. repetitive sequences) or <5X per allele (inadequate depth), SNPs in Hardy-Weinberg disequilibrium and, as requested by Reviewer 1, SNPs overlapping blacklisted regions. In addition, SNPs where none of the reads contained both the index SNPs and at least 3 CpGs were filtered out. This last filtering step in fact represents most of the filtered SNPs. While it will be interesting to study the mechanism of bona fide “single CpG ASM”, sets of apparent single CpG ASM are likely to be more contaminated by false-positive ASM calls, so we feel that this type of analysis should be undertaken as a separate study.*

*Lastly, while the set of Agilent informative SNPs is biased toward regulatory elements, its small proportions did not significantly bias our main findings, as confirmed by our careful sub-analysis using only WGBS samples from RCPI facility (Fig. S12 of the MS and Response Figures I and II here).*

Q4:	Apparently, because genomes have not been sequenced, only the "index hets" involving minor alleles with relatively high frequency have been assessed. How did this bias away from variants that have large effects and therefore tend to have low frequency due to purifying selection affected analyses?

*As explained in Q3, we did not pre-select the index SNPs. Therefore theoretically, since we included 75 different individuals, the informative SNPs would have an allele frequency of >1.5% to be seen in at least one sample and 3% to be seen in at least in 2 samples (required in our analysis). The allele frequencies of our informative SNPs are in fact similar to dbSNP allele frequencies. Although our set is (of necessity) still biased toward common SNPs (>1%), our study included many different individuals, which reduces this bias.*

Q5:	The "index hets" are identified by intersecting WGBS data with a list of SNPs from the public domain. How was the list of SNPs compiled?

*See answer to Q3.*

Q6:	How was the bias toward better mapping of reads with major alleles (reference sequence bias) been addressed for allele-specific ChIP-seq (p.15-16).

*To minimize technical bias toward one allele (mapping or PCR bias), the random expectation for allele distribution in a ChIP-seq peak is 50% for both alleles and we defined ASB as when the frequency of one allele was markedly higher, namely > 80%.*

Q7:	Could the absence of "reverse bias" where the disruption of TF binding decreases methylation be explained by any ascertainment differences between the present and previous study? If so, what are the potentially most relevant ones?

*In the current study, we found 482 TFBS motifs with positive ASM correlation trends. However, in contrast to the numerous TF motif classes that show strong and highly significant inverse correlations in our study (Table S8), only 2 of the examples with positive (not inverse) correlations pass our stringent correlation criteria (FDR<0.05 and Rsq>0.4).*

*Compared to our previous study, we tested a larger set of motifs (2500), therefore for a small subset of 15 motifs which did not pass the multiple testing correction but show a relatively good Rsq (>0.2), the low number of occurrences for these motifs explains a lack of power. However, for most of these motifs the Rsq was low <0.2 , suggesting that TF motifs with positive correlations show more heterogeneity (inter-tissue or inter-individual). See our working hypothesis models in Fig S18.*

Q8:	Is there evidence that the ASM for GWAS regulatory SNPs associates with allelic transcription in cis in the etiologically relevant cell types?

*We agree this aspect is important, so we have added in Tables S10-S12 annotations of the ASM SNPs for eQTL SNPs in LD with immune, cancer or neuro-psychiatric GWAS peaks where allelic transcription imbalance was observed in a relevant organ (blood, brain, lymphoblastoid cell lines) using the GRASP database. For GWAS SNPs associated with cancer, we report eQTLs from lymphoblastoid cell lines since the GRASP database does not include data on cancer eQTLs. In addition, we report eQTLs reported in blood cells, since immune system cells are relevant to cancer immune surveillance.*

Q9:	p.5, Line 52: Which glia and neuron samples are being referred to? The type of GBM (if CIMP subtype, it should be stated as it is relevant for interpreting DNA methylation results).

*This refers to the NeuN-negative (glia) and NeuN-positive (neurons) samples that we prepared by FANS from non- cancer autopsy brains, as listed in Table S1. We agree that GBM sub-typing for the CpG island methylator phenotype (CIMP) is of interest. Our current series (6 cases) is too small for definite conclusions about the relationship of CIMP to ASM, but we have added some preliminary findings in the updated Results section.*

Q10:	p.5 First paragraph: Unclear phrase: "non-CpG containing polymorphic CTCF motif instances".

*We updated our manuscript to clarify this sentence.*

Q11:	p.8, Line 20-25: Unclear statement.

*We updated our manuscript to clarify this sentence.*

*The term "Human Epigenome Project" was used as an incorrect reference to the "NIH Roadmap Epigenomics Project"*

*We thank the reviewer to point this incorrect reference and revised our manuscript adding the correct one.*

Q12: p.14. Lines 43-48: How many motifs are found in the randomized set, compared to the 860 found in real data? This number will help interpret the result if spelled out in the main text.

*Out of 2608 tested TF motifs, 2663 had >10 occurrences in the randomized set or ASM set (we did not request 10 occurrences in both sets, so we would be able to assess TF motif depletion)s and were tested for enrichment. Using this approach, 38% of the TF motifs showed an enrichment among ASM. We have specified these numbers in the revised MS.*

*Supplemental Excel tab "S2 ASM All excludes imprinted" shows more than 13,200 ASM SNPs whereas the paper reports less than 13,000; why the difference?*

*Each line of our Table S2 shows an index SNP associated with ASM (i.e. the SNPs are the unique identifiers). For some SNP dense regions, multiple SNPs in the same contig are associated with a single ASM DMR. Therefore, we provided a “DMR” column to tag the unique region queried by these multiple SNPs, and in our MS we report both the number of ASM SNPs and the number of DMRs.*

*In the supplemental spreadsheet, "rsIDs" refer to polymorphisms, not to individual alleles. Some important allele-specific information (e.g., methylation levels in specific WGBS datasets) may be communicated by reference to specific alleles (e,g., using the ClinGen Allele Registry to obtain the IDs of specific alleles).*

*We only included biallelic SNPs to call ASM using the reference and alternate allele reported in DbSNP 147. We added columns to report the reference and alternate allele used in our ASM call. The averaged methylation level for each of these alleles (REF and ALT) are reported in Table S2. Allelic imbalances found in our study (methylation difference, binding affinity) are reported comparing the ALT allele to the REF allele.*

**Second round of review**

**Reviewer 1**

The authors have thoroughly responded to all my concerns and added new results in a satisfactory manner. I have no further comments.

**Reviewer 2**

The paper will benefit from streamlining to eliminate complex and less descriptions of less widely understood patterns of correlations to better communicate with the wide audience of Genome Biology.

Consider moving some figures/panels into Supplemental materials (e.g., panels 4D, 6C).

Legend in Fig1 panel B is redundant with Panel A Legend.

Do the figure numbers match captions (after insertion of new Figure 5)?

“Nb.” Is an unusual abbreviation for “Number”. A Hashtag or “Num.” will be better understood.

**Authors Response**

**Point-by-point responses to the reviewers’ comments:**

Reviewer #2: The paper will benefit from streamlining to eliminate complex and less descriptions of less widely understood patterns of correlations to better communicate with the wide audience of Genome Biology.

Consider moving some figures/panels into Supplemental materials (e.g., panels 4D, 6C).

*Panel 4D has been moved to Suppl.16A. We feel that Fig. 6 is best left intact with the original panels.*

Legend in Fig1 panel B is redundant with Panel A Legend.

*Legend of panel B has been trimmed to avoid redundancy.*

Do the figure numbers match captions (after insertion of new Figure 5)?

*Yes in the text but the new figure 5 was labeled by mistake figure 6 in the PDF. We fixed this.*

*“Nb.” Is an unusual abbreviation for “Number”. A Hashtag or “Num.” will be better understood.*

*We replaced Nb. by a hashtag.*

*NOTE: we have found one more bona fide imprinted region that was previously listed in Table S5 as a “possibly imprinted” region. Accordingly, we have moved this region (2 ASM SNPs and 2 ASM DMRs) to Table S4 and corrected the numbers of non-imprinted ASM regions in the Abstract and body of our text. Thus:*

*“After excluding imprinting, the data pinpoint 15,112 (corrected from 15,114) high-confidence ASM differentially methylated regions, of which 1,838 contain SNPs in strong linkage disequilibrium or coinciding with GWAS peaks.”*
